# Supplementary material for: Metabolic syndrome promotes endometrial cancer by Oleic acid-mediated polyamine accumulation
Source: Nat Commun. 2025 Dec 16;17:388. doi: 10.1038/s41467-025-67083-y (PMC12796254; doi:10.1038/s41467-025-67083-y)
Supplement: Supplementary file 1 — Supplementary Information File [file 41467_2025_67083_MOESM1_ESM.pdf]

Supplementary Materials for

Metabolic Syndrome Promotes Endometrial Cancer by Oleic Acid-Mediated Polyamine Accumulation

Lirong Zhai *et al.*

* Jianliu Wang. Email: wangjianliu@pkuph.edu.cn
* Jun Zhan. Email: zhanjun@bjmu.edu.cn

This PDF file includes:

Figs. S1 to S11
Tables S1 to S12

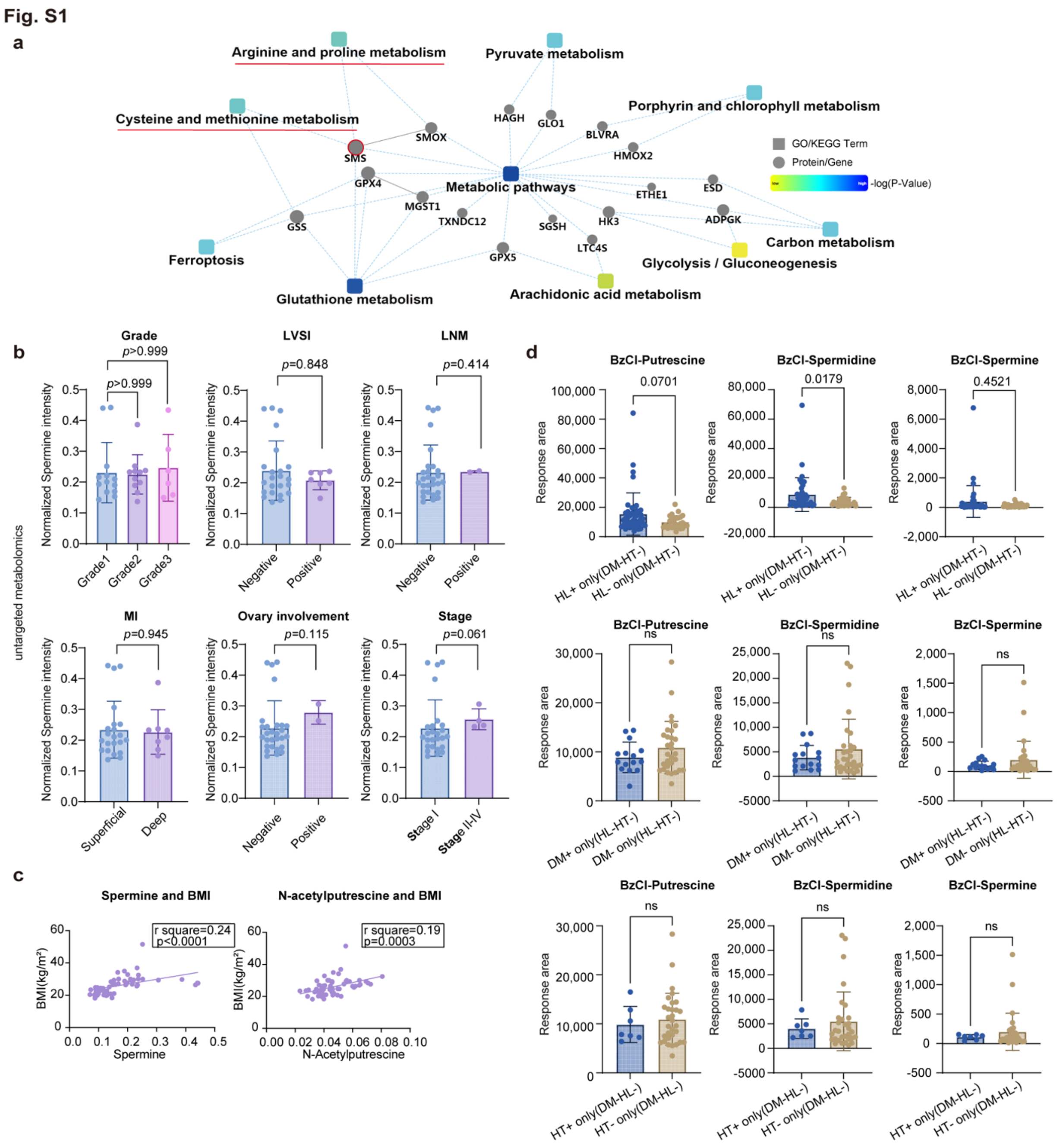

Fig. S1. Polyamines were elevated in EC patients with hyperlipidemia but not diabetes mellitus and hypertension (related to Figure 1)

(a). Metabolic enzyme association analysis. Gray squares: GO/KEGG terms; gray circles: proteins/genes; color gradient (yellow to blue): low to high -log(P-Value). (b) Differences of normalized spermine intensity (untargeted metabolomics) across pathological factors (grade, lymph node metastases [LNM], lymph vascular space invasion [LVSI], myometrial invasion [MI], ovarian involvement, stage) in the MS+ Group (N=30) of the identification cohort. Two-tailed Mann-Whitney U test (two groups); Two-tailed Kruskal-Wallis test (multiple groups).

(c). Correlation of spermine and N-acetyl putrescine with Body Mass Index (BMI) in the identification cohort (N=62). Data: mean ± SD. *p < 0.05, **p < 0.01, ***p < 0.001, ****p < 0.0001 Two-tailed Pearson correlation test. (d). Response areas of Benzoyl chloride (BzCl)-derivatized putrescine, spermidine, and spermine in validation cohort patients (N=156) with/without hyperlipidemia (HL), diabetes mellitus (DM), or hypertension (HT). Data: mean ± SD. *p < 0.05, **p < 0.01, ***p < 0.001, ****p < 0.0001 Two-tailed Mann Whitney test. Source data are provided as a Source Data file. Exact p-values are provided in the Source Data file.

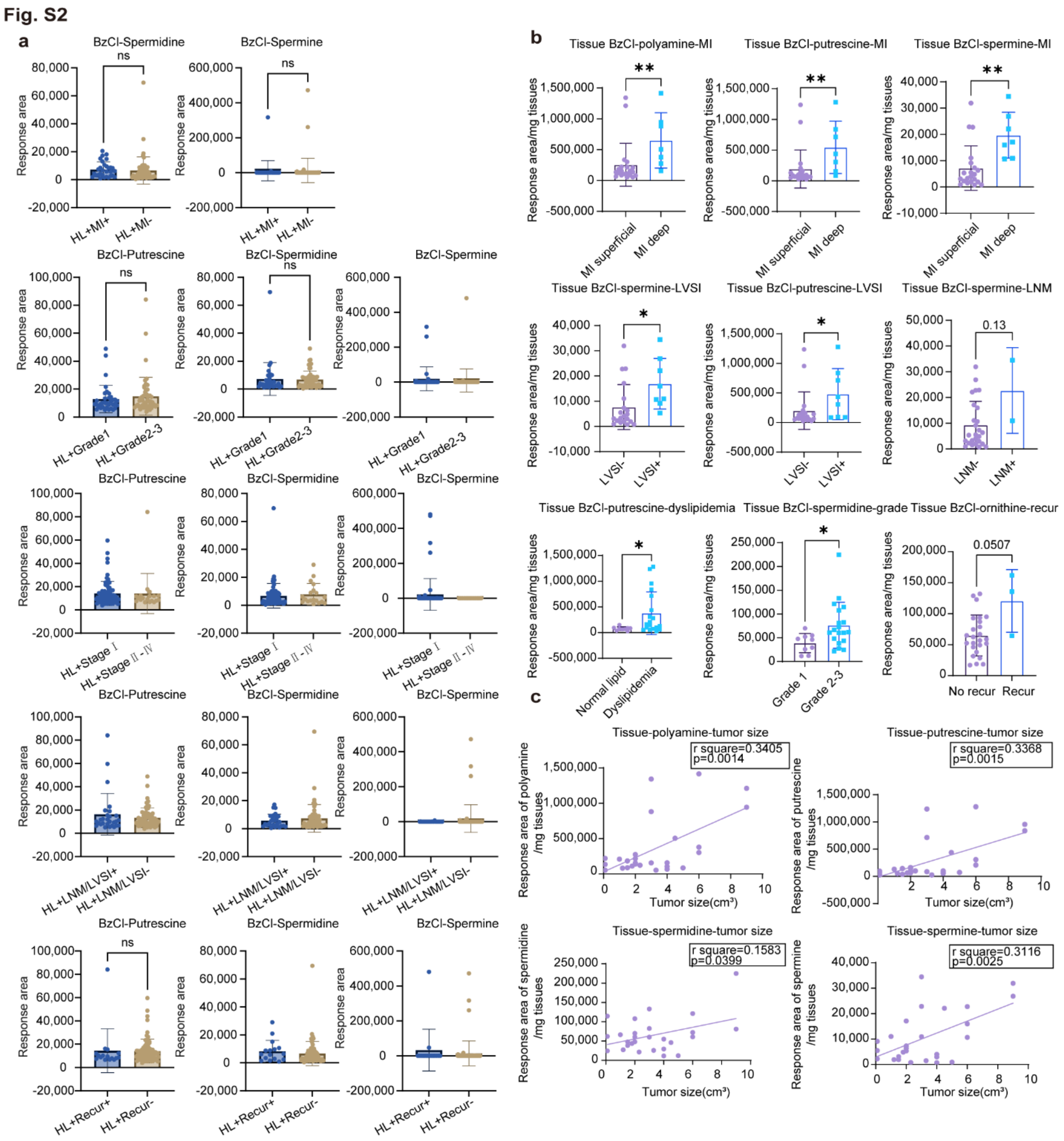

Fig. S2. Tissue polyamines were associated with tumor invasiveness and tumor size. (related to Figure 1)

(a) Response areas of BzCl-derivatized putrescine, spermidine, and spermine in sera of hyperlipidemia (HL) patients (validation cohort, N=92) stratified by myometrial invasion (MI), grade, stage, lymph node metastases/ lymph vascular space invasion (LNM/LVSI), or recurrence status. Data: mean ± SD. *$p < 0.05$, **$p < 0.01$, ***$p < 0.001$, ****$p < 0.0001$ Two-tailed Mann Whitney test. (b) Response areas of BzCl-derivatized putrescine, spermidine, spermine, and ornithine in tumor tissues (identification cohort) stratified by MI, LVSI, LNM, grade, recurrence,

or hyperlipidemia status. Data: mean ± SD. $*p < 0.05$, $**p < 0.01$, $***p < 0.001$, $****p < 0.0001$ Two-tailed Mann Whitney test. (c) Correlation of BzCl-derivatized putrescine, spermidine, and spermine levels in tumor tissues with tumor size (identification cohort). Two-tailed Pearson correlation test. Source data are provided as a Source Data file. Exact p-values are provided in the Source Data file.

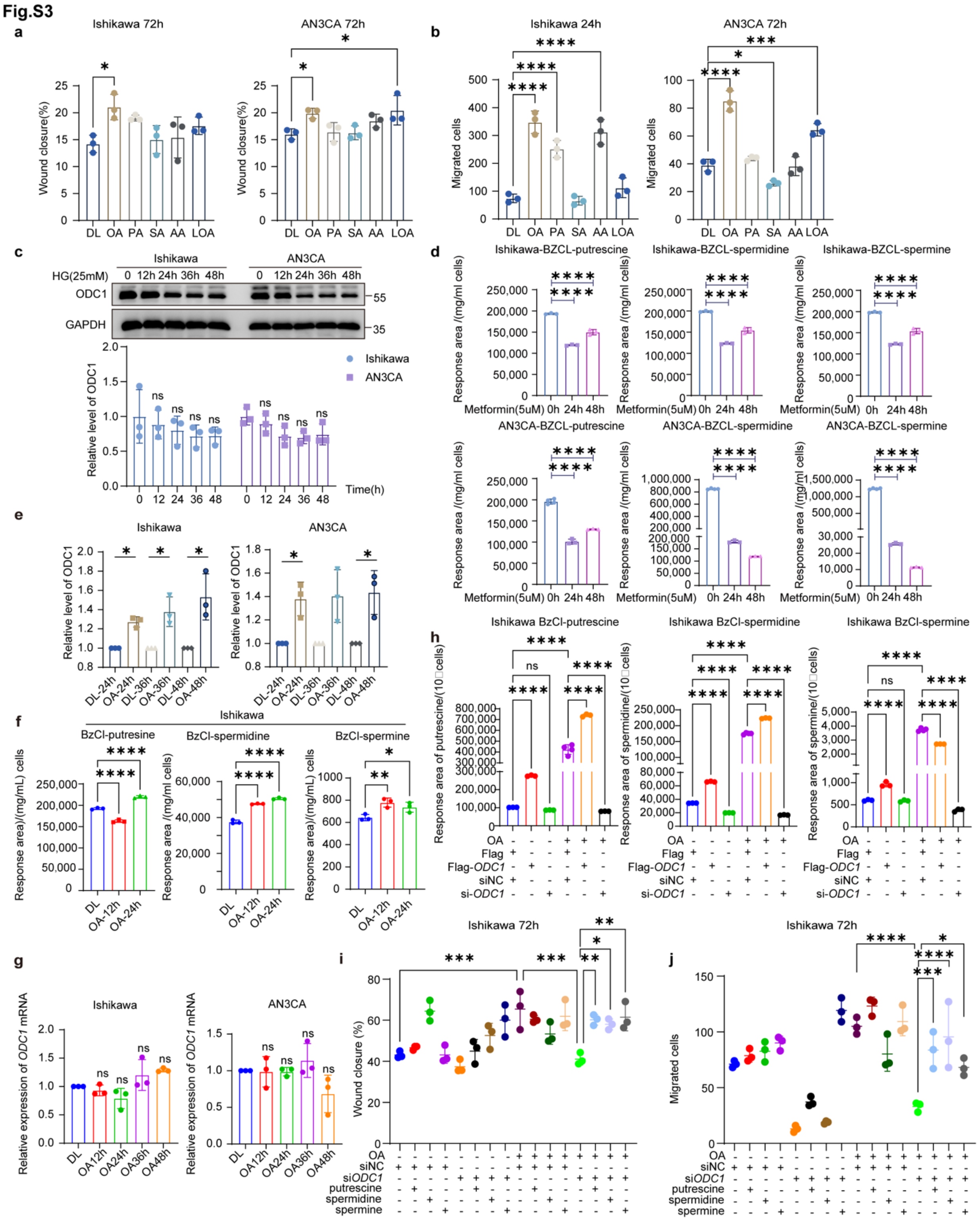

Fig. S3. OA in hyperlipidemia upregulated ODC1-polyamine metabolism, promoting EC progression (related to Figure 2)

(a) Wound healing assay for Ishikawa/AN3CA cell migration (various free fatty acids [FFAs]: delipidated [DL], oleic acid [OA], palmitic acid [PA], Stearic Acid [SA], Arachidonic acid [AA],

Linoleic acid [LOA]; 30μM, 72h). Data: mean ± SEM (n=3 independent. experiments). *p<0.05, **p<0.01, ***p<0.001, ****p<0.0001 (Two-tailed One-way ANOVA). (b) Transwell assay for cell migration (various FFAs, 30μM, 24/72h). Data: mean ± SEM (n=3 independent. experiments). *p<0.05, **p<0.01, ***p<0.001, ****p<0.0001 (Two-tailed One-way ANOVA). (c) Upper: WB of whole-cell lysates (high glucose [HG], 4.5g/L, indicated hours). Lower: ODC1 band grayscale quantification. Data: mean ± SEM (n=3 independent. experiments). *p<0.05, **p<0.01, ***p<0.001, ****p<0.0001 (Two-tailed One-way ANOVA). (d) LC-MS of BzCl-derivatized polyamines (metformin, 5μM, indicated times). Data: mean ± SD (n=3 technical replicates; trend in 3 independent. experiments). *p<0.05, **p<0.01, ***p<0.001, ****p<0.0001 (Two-tailed One-way ANOVA). (e) ODC1 grayscale quantification (OA, 30μM, indicated times; Fig. 2g). Data: mean ± SEM (n=3 independent. experiments). Two-tailed Student's t-test. (f) LC-MS of BzCl-derivatized polyamines (Ishikawa cells, OA, 30μM, indicated times). Data: mean ± SD (n=3 technical replicates; trend in 3 independent. experiments). *p<0.05, **p<0.01, ***p<0.001, ****p<0.0001 (Two-tailed One-way ANOVA). (g) *ODC1* mRNA expression (OA, 30μM, indicated times). Data: mean ± SEM (n=3 independent. experiments). *p<0.05, **p<0.01, ***p<0.001, ****p<0.0001 (Two-tailed One-way ANOVA). (h) LC-MS of BzCl-derivatized polyamines (Ishikawa cells: ±OA, 30μM; transfected with siNC/si*ODC1*/Flag/Flag-*ODC1*, 48h; OA added 24h pre-harvest). Polyamine content: response area normalized to cell number. Data: mean ± SD (n=3 technical replicates; trend in 3 independent. experiments). *p<0.05, **p<0.01, ***p<0.001, ****p<0.0001 (Two-tailed One-way ANOVA). (i) Wound healing assay (Ishikawa cells: ±OA, 30μM; siNC/si*ODC1*; ±putrescine 50μM/spermidine 10μM/spermine 10μM, 72h; OA added 24h pre-harvest). Data: mean ± SEM (n=3 independent. experiments). *p<0.05, **p<0.01, ***p<0.001, ****p<0.0001 (Two-tailed One-way ANOVA). (j) Transwell assay (same treatment as (i)). Data: mean ± SEM (n=3 independent. experiments). *p<0.05, **p<0.01, ***p<0.001, ****p<0.0001 (Two-tailed One-way ANOVA). Multiple comparisons were corrected. Source data are provided as a Source Data file. Exact p-values are provided in the Source Data file.

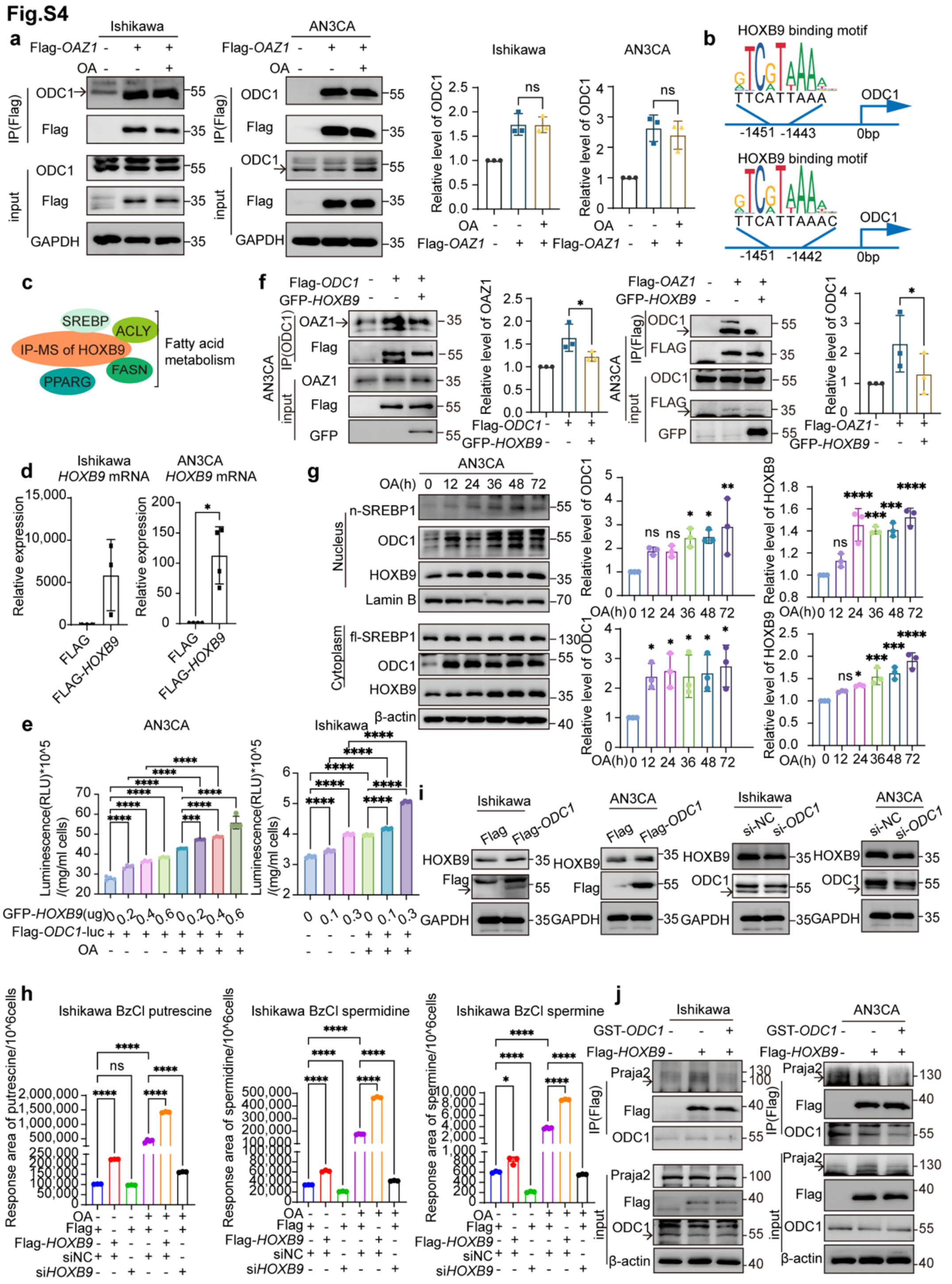

Fig. S4. OA does not affect the binding of ODC1 to OAZ1 (related to Figure 3)

(a) Co-IP (Ishikawa/AN3CA cells: Flag/Flag-*OAZ1*/Flag-*OAZ1*+OA [30µM], anti-Flag beads) + WB (anti-ODC1). Right: ODC1 blot grayscale quantification. Data: mean ± SEM (n=3 independent experiments). *p < 0.05, **p < 0.01, ***p < 0.001, ****p < 0.0001 (Two-tailed Ratio paired t-test). Same-experiment samples on different gels (ODC1, Flag; ODC1, GAPDH),

all processed in parallel. (b) Prediction of HOXB9-ODC1 promoter binding site (JASPAR: https://jaspar.elixir.no/). (c) Immunoprecipitation-Mass Spectrometry (IP-MS) of HOXB9[37]: lipid metabolism-related protein enrichment. (d) *HOXB9* mRNA of Ishikawa/AN3CA cells transfected with Flag/Flag-*HOXB9*. Data: mean ± SEM (⩾3 independent experiments). *p < 0.05, **p < 0.01, ***p < 0.001, ****p < 0.0001 (Two-tailed Student's t-test). (e) Ishikawa/AN3CA cells: Flag-*ODC1*-luc (0.25μg) + GFP-*HOXB9* (0.1–0.6μg) ± OA (30μM). Luciferase activity was normalized to protein concentration. Data: mean ± SD (n=4 wells; trend in 3 independent experiments). *p < 0.05, **p < 0.01, ***p < 0.001, ****p < 0.0001 (Two-tailed One-way ANOVA). (f) Left: Co-IP (AN3CA cells: GFP-*HOXB9*+Flag-*ODC1*, anti-ODC1 beads) + WB (anti-OAZ1) + quantification. Right: Co-IP (GFP-*HOXB9*+Flag-*OAZ1,* anti-Flag beads) + WB (anti-ODC1) + quantification. Data: mean ± SEM (n=3 independent experiments). *p < 0.05, **p < 0.01, ***p < 0.001, ****p < 0.0001 (Two-tailed Ratio paired t-test). Same-experiment samples, different gels: left (OAZ1, Flag; GFP), right (ODC1, Flag; GFP) gels, all processed in parallel. (g) Left: HOXB9/ODC1/SREBP1 levels (AN3CA cytoplasmic/nuclear lysates, OA [30μM], indicated times). Right: ODC1/HOXB9 quantification. Data: mean ± SEM (n=3 independent experiments). *p < 0.05, **p < 0.01, ***p < 0.001, ****p < 0.0001 (Two-tailed One-way ANOVA). Same-experiment samples, different gels: nucleus (n-SREBP1, HOXB9; Lamin B, ODC1), cytoplasm (fl-SREBP1, HOXB9; β-actin, ODC1), all processed in parallel. (h) LC-MS (BzCl-derivatized polyamines, Ishikawa cells: ±OA [30μM] + siNC/si*HOXB9*/Flag/Flag-*HOXB9*, 48h). Data: mean ± SD (n=3 technical replicates; trend in 3 independent experiments). *p < 0.05, **p < 0.01, ***p < 0.001, ****p < 0.0001 (Two-tailed One-way ANOVA). (i) HOXB9 protein (Ishikawa/AN3CA cells: Flag/Flag-*ODC1*/siNC/si*ODC1*, 48h). n=3 independent experiments. Same-experiment samples on different gels (ODC1/Flag, GAPDH; HOXB9), processed in parallel. (j) Co-IP (Ishikawa/AN3CA cells: GST-*ODC1*+Flag-*HOXB9*, anti-Flag beads) + WB (anti-Praja2). n=3 independent experiments. Same-experiment samples on different gels (Praja2, ODC1, Flag; Flag, β-actin), processed in parallel. Multiple comparisons were corrected. Source data are provided as a Source Data file. Exact p-values are provided in the Source Data file.

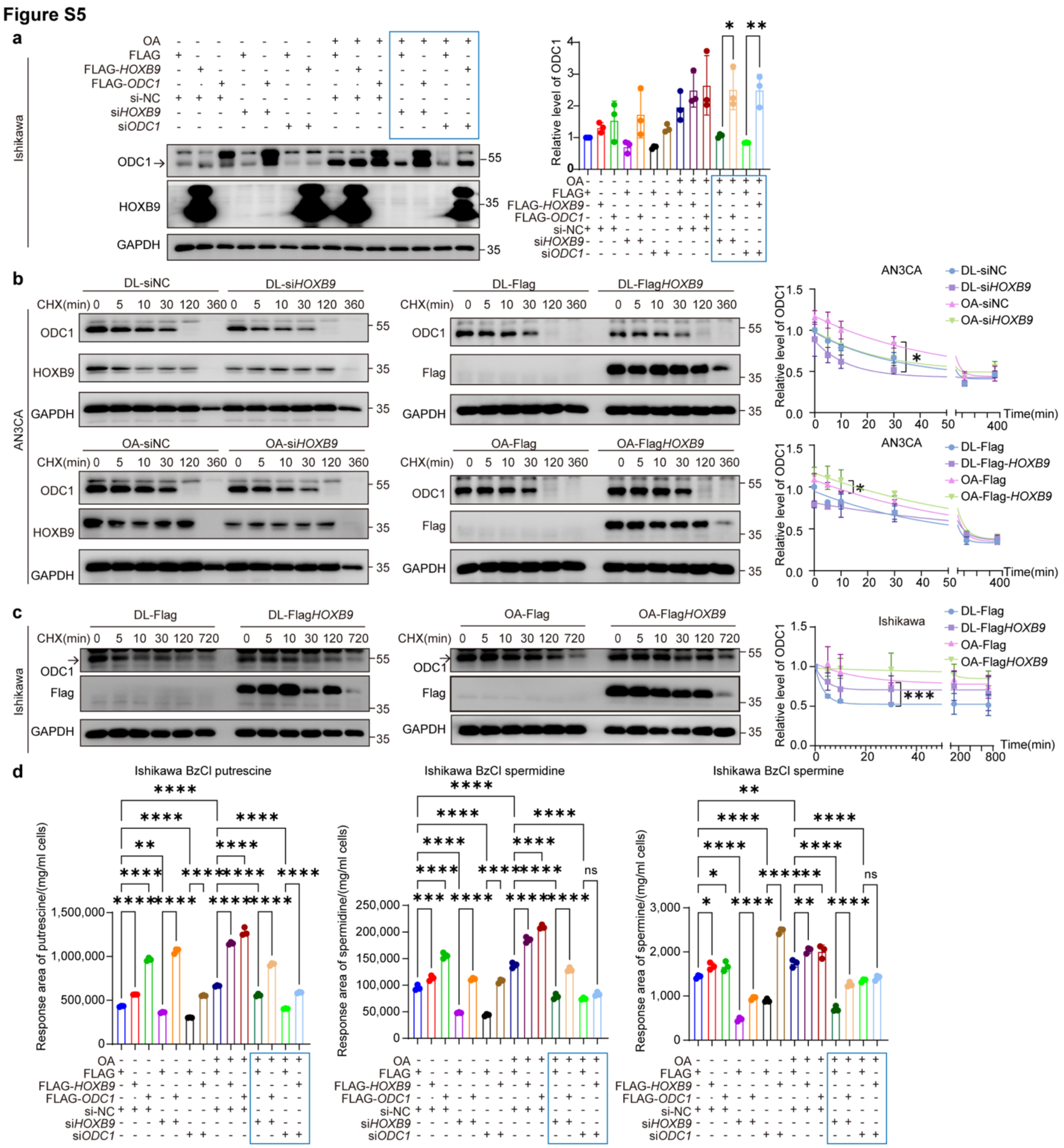

Fig. S5. HOXB9-ODC1 is required for OA induced polyamine accumulation (related to Figure 4)

(a) ODC1 and HOXB9 protein levels (WB) in Ishikawa cells: treated with/without OA (30μM), transfected with si*NC*, si*HOXB9*, si*ODC1*, Flag, Flag-*HOXB9*, or Flag-*ODC1* for 72h (OA added 24h before harvest) Data: mean ± SEM (n=3 independent experiments). *$p < 0.05$, **$p < 0.01$, ***$p < 0.001$, ****$p < 0.0001$ (Two-tailed One-way Anova). Boxed areas indicate key rescue groups: si*ODC1* + Flag-*HOXB9* and si*HOXB9* + Flag-*ODC1*. Samples from the same experiment used different gels (ODC1, GAPDH; HOXB9), all processed in parallel. (b) AN3CA cells were treated with or without OA (30μM) and transfected with siNC, si*HOXB9*, Flag, or

Flag-*HOXB9* for 48 hours. CHX (100µg/mL) was added at different time points prior to protein harvesting. ODC1 protein levels were detected by WB. Right: ODC1 degradation curve. Data: mean ± SEM (n=3 independent experiments). $*p < 0.05$, $**p < 0.01$, $***p < 0.001$, $****p < 0.0001$ Two-tailed One-way ANOVA. Samples from the same experiment used different gels (ODC1, GAPDH; HOXB9/Flag), all processed in parallel. (c). Ishikawa cells were treated with or without OA (30µM) and transfected with Flag or Flag-*HOXB9* for 48 hours. CHX (100µg/mL) was added at different time points prior to protein harvesting. ODC1 protein levels were detected by WB. Right: ODC1 degradation curve. Data: mean ± SEM (n=3 independent experiments). $*p < 0.05$, $**p < 0.01$, $***p < 0.001$, $****p < 0.0001$ Two-tailed One-way ANOVA. Samples from the same experiment used different gels (ODC1, GAPDH; Flag), all processed in parallel. (d). LC-MS of BzCl-derivatized putrescine, spermidine, and spermine in Ishikawa cells: treated with/without OA (30µM) and transfected with siNC, si*HOXB9*, si*ODC1*, Flag, Flag-*HOXB9*, or Flag-*ODC1* for 72 hours (OA added 24h before harvest). Data: mean ± SD (n = 3 technical replicates; trend detected in 3 independent experiments). $*p < 0.05$, $**p < 0.01$, $***p < 0.001$, $****p < 0.0001$ Two-tailed One-way ANOVA. Boxed areas indicate key rescue groups: si*ODC1* + Flag-*HOXB9* and si*HOXB9* + Flag-*ODC1*. Multiple comparisons were corrected. Source data are provided as a Source Data file. Exact p-values are provided in the Source Data file.

Fig.S6

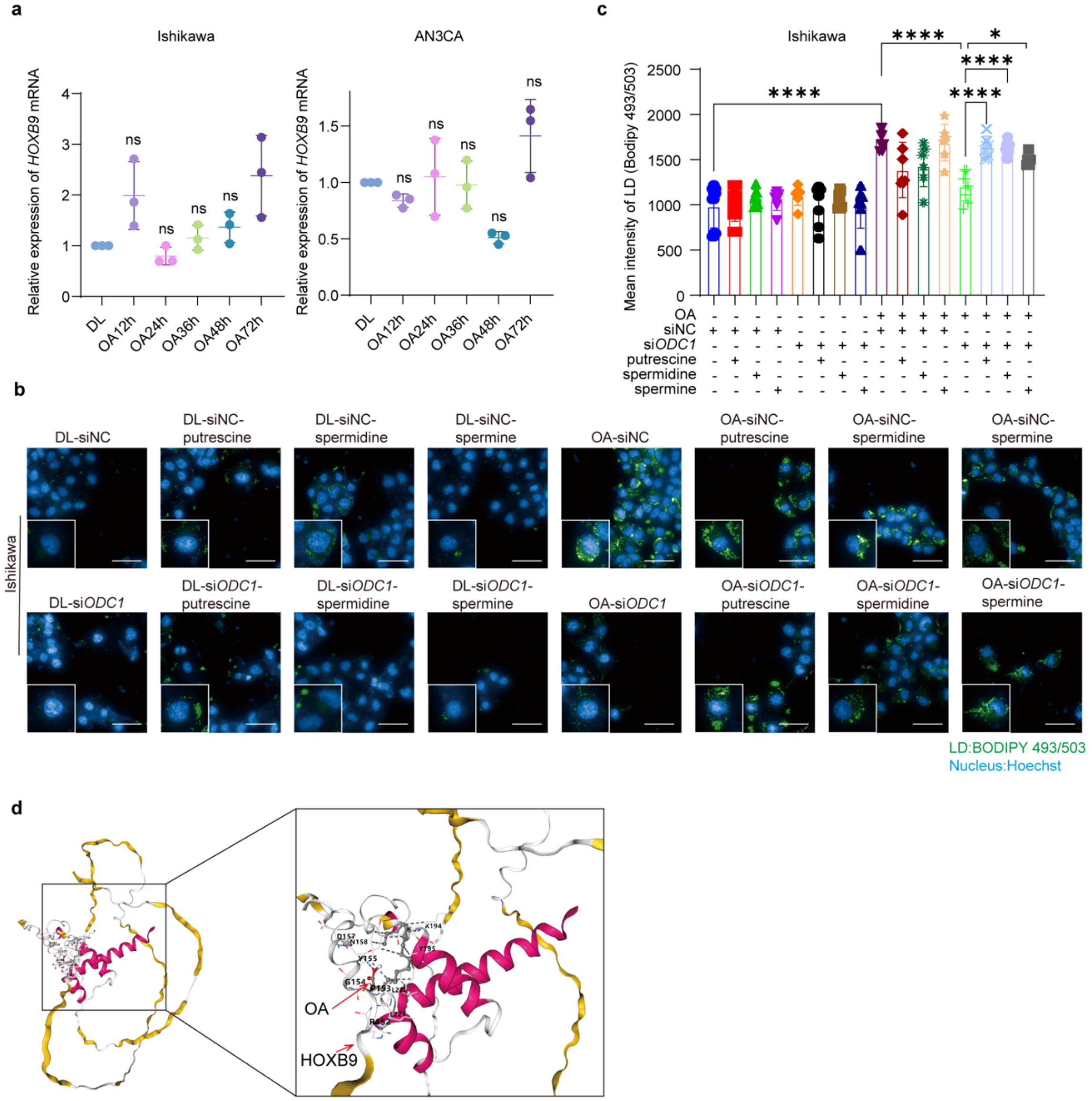

Fig. S6. OA did not regulate HOXB9 at the transcription level (related to Figure 5)

(a). *HOXB9* mRNA levels in Ishikawa and AN3CA cells treated with OA (30μM) for indicated hours. Data: mean ± SEM (n=3 independent experiments). *p < 0.05, **p < 0.01, ***p < 0.001, ****p < 0.0001 Two-tailed One-way ANOVA. (b). Representative Bodipy 493/503 staining of lipid droplets in Ishikawa cells from 3 independent experiments: treated with/without OA (30μM), transfected with siNC or si*ODC1*, supplemented with/without putrescine (50μM), spermidine (10μM), or spermine (10μM) for 72h. Scale bars: 100 μm. (c). Statistical analysis of mean lipid droplet intensity in (b). Data: mean ± SD (counted in 3–6 fields; trend observed in 3 independent experiments). *p < 0.05, **p < 0.01, ***p < 0.001, ****p < 0.0001 Two-tailed One-way ANOVA. (d). Molecular docking of OA (SMILE: SCCCCCCCCC=CCCCCCCCC(=O)O) and HOXB9 (https://alphafold.ebi.ac.uk/entry/P17482) by CB-DOCK2

(https://cadd.labshare.cn/cb-dock2). Source data are provided as a Source Data file. Exact p-values are provided in the Source Data file.

Fig. S7

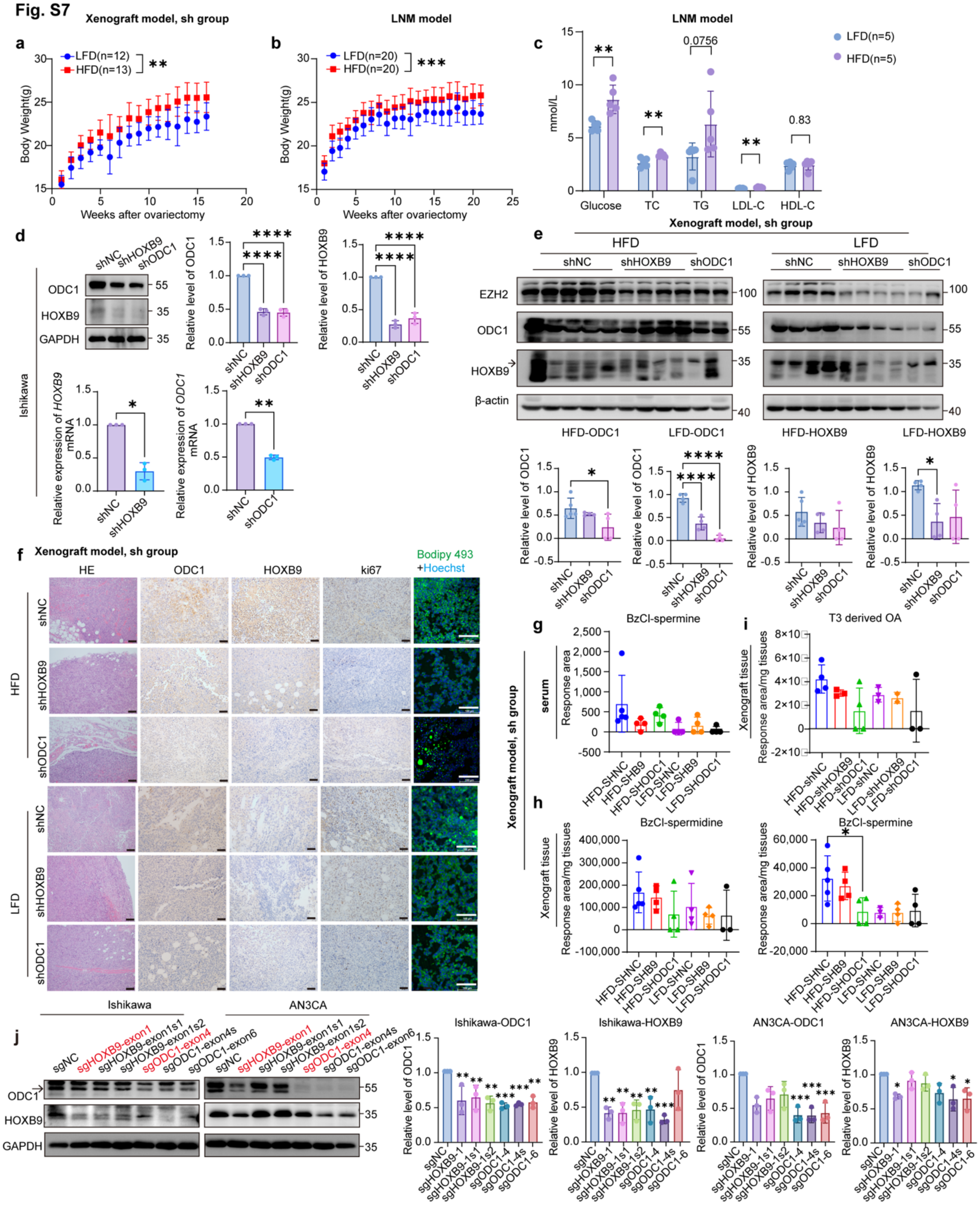

Xenograft model, sh group
LNM model
LNM model
a
b
c
LFD(n=12)
HFD(n=13)
LFD(n=20)
HFD(n=20)
LFD(n=5)
HFD(n=5)
Body Weight(g)
Weeks after ovariectomy
mmol/L
Glucose
TC
TG
LDL-C
HDL-C
0.0756
0.83
Xenograft model, sh group
d
e
HFD
LFD
shNC
shHOXB9
shODC1
ODC1
HOXB9
GAPDH
Ishikawa
EZH2
β-actin
Relative level of ODC1
Relative level of HOXB9
Relative expression of HOXB9 mRNA
Relative expression of ODC1 mRNA
HFD-ODC1
LFD-ODC1
HFD-HOXB9
LFD-HOXB9
f
Xenograft model, sh group
HE
ODC1
HOXB9
ki67
Bodipy 493
+Hoechst
g
BzCl-spermine
serum
Response area
i
T3 derived OA
Xenograft tissue
Response area/mg tissues
h
BzCl-spermidine
BzCl-spermine
Xenograft model, sh group
HFD-SHNC
HFD-SHB9
HFD-SHODC1
LFD-SHNC
LFD-SHB9
LFD-SHODC1
j
Ishikawa
AN3CA
sgNC
sgHOXB9-exon1
sgHOXB9-exon1s1
sgHOXB9-exon1s2
sgODC1-exon4
sgODC1-exon4s
sgODC1-exon6
Ishikawa-ODC1
Ishikawa-HOXB9
AN3CA-ODC1
AN3CA-HOXB9
sgHOXB9-1
sgHOXB9-1s1
sgHOXB9-1s2
sgODC1-4
sgODC1-4s
sgODC1-6

Fig. S7. Knocking-down HOXB9 and ODC1 inhibit Ki-67 expression and lipid droplet formation in xenografts of HFD induced EC mice model (related to Figure 6)

(a) Subcutaneous xenograft model (sh group): mouse weight curves (LFD: n=12; HFD: n=13, experiment end). Data: mean ± SD. Two-tailed Two-way ANOVA. (b) Footpad injection lymph node metastasis (LNM) model: mouse weight curves (LFD/HFD: n=20 each, experiment end). Data: mean ± SD. Two-way ANOVA. (c) LNM model: serum glucose/TG/TC/LDL-c/HDL-c (2-month diet induction, Low-fat diet/ High-fat diet [LFD/HFD]: n=5 each). Data: mean ± SD. Two-tailed Student's t-test. (d) Ishikawa cells (stable transfection: shNC/sh*HOXB9*/sh*ODC1*): Upper: ODC1/HOXB9 protein (WB) + right: band grayscale stats; Lower: *ODC1/HOXB9* mRNA. Data: mean ± SEM (n=3 independent experiments). Two-tailed One-way ANOVA for protein; Student's t-test for RNA. Same-experiment samples on different gels (ODC1, GAPDH; HOXB9), processed in parallel. (e) Subcutaneous xenograft model (sh group): xenograft lysate HOXB9/ODC1/EZH2 (HFD-NC: n=5, others: n=4 per group). Lower: ODC1/HOXB9 grayscale. No tumor: WB signal=0. Data: mean ± SD. Two-tailed One-way ANOVA. Same-experiment samples on different gels (EZH2, ODC1; HOXB9, β-actin), processed in parallel. (f) Subcutaneous model: xenograft IHC (HOXB9/ODC1/Ki67) + Bodipy 493/503 (lipid droplets; HFD-NC: n=5, others: n=4 per group). Scale bars: 100 μm (HE), 50 μm (IHC), 100 μm (Bodipy). (g) Subcutaneous model: serum BzCl-derivatized spermine (HFD-NC: n=5, others: n=4 per group). Data: mean ± SD. Two-tailed One-way ANOVA. (h) Subcutaneous model: xenograft BzCl-derivatized spermidine/spermine (HFD-NC: n=5, others: n=4 per group). No tumor: response=0. Data: mean ± SD. Two-tailed One-way ANOVA. (i) Subcutaneous model: xenograft T3-derivatized OA (group n: HFD-NC=4, HFD-shHOXB9=3, HFD-shODC1=4, LFD-NC=3, LFD-shHOXB9=2, LFD-shODC1=3). For mice without tumor formation, the response signal was recorded as 0; for samples with insufficient tissue quantity, the data were recorded as "not enough tissue". Data: mean ± SD. Two-tailed One-way ANOVA. (j) Ishikawa/AN3CA cells: sgRNA-based HOXB9/ODC1 knockdown (different exons) validation (red indicates HOXB9-exon1/ODC1-exon4 selected). Right: ODC1/HOXB9 grayscale. Data: mean ± SEM (n=3 independent experiments). Two-tailed One-way ANOVA. Same-experiment samples on different gels (ODC1, GAPDH; HOXB9), processed in parallel.

*$p < 0.05$, **$p < 0.01$, ***$p < 0.001$, ****$p < 0.0001$. Multiple comparisons were corrected. Source data are provided as a Source Data file. Exact p-values are provided in the Source Data file.

Fig. S8

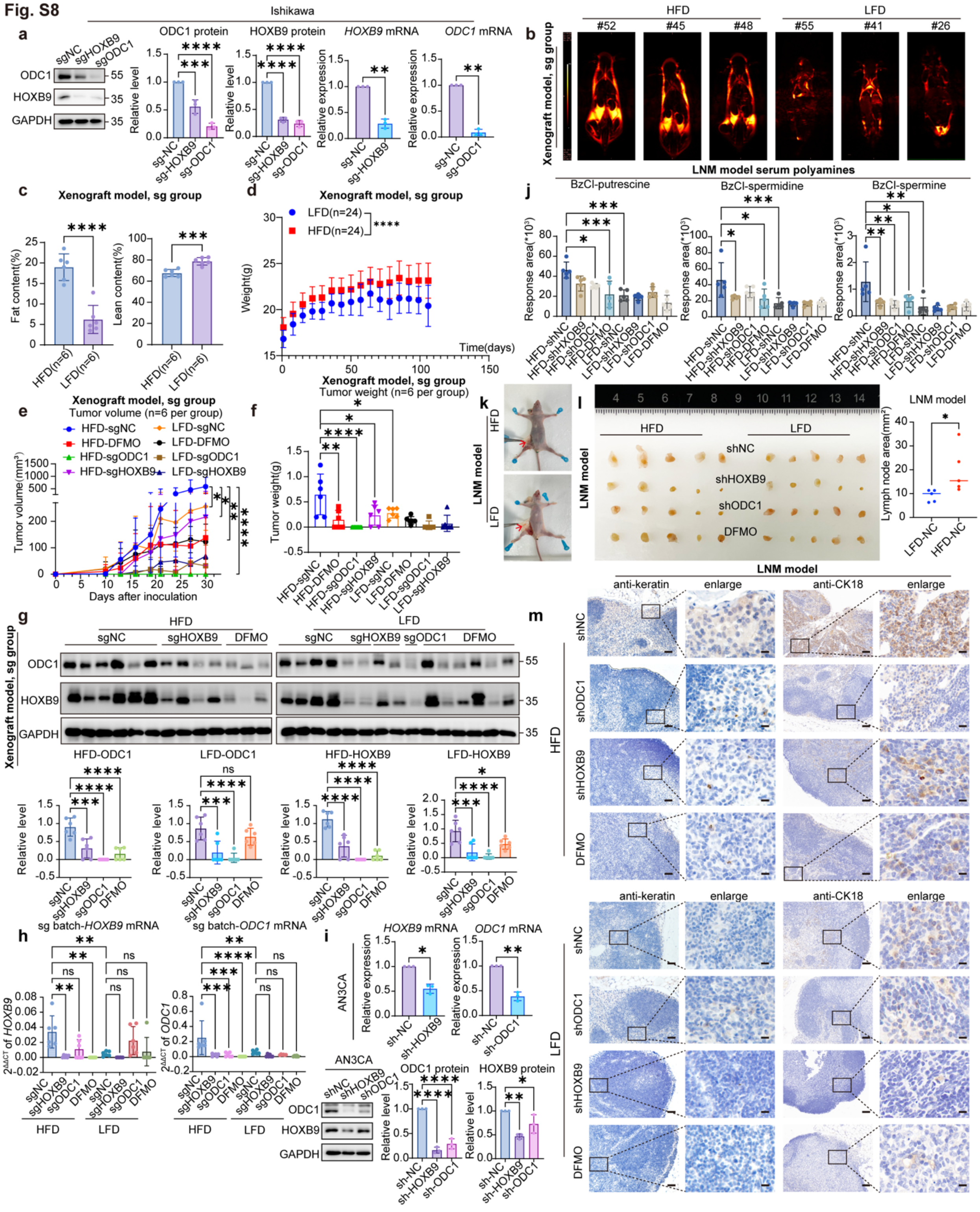

Ishikawa
ODC1 protein
HOXB9 protein
HOXB9 mRNA
ODC1 mRNA
HFD
LFD
Xenograft model, sg group
LNM model serum polyamines
BzCl-putrescine
BzCl-spermidine
BzCl-spermine
Tumor volume (n=6 per group)
Tumor weight (n=6 per group)
Days after inoculation
LNM model
anti-keratin
enlarge
anti-CK18
sg batch-HOXB9 mRNA
sg batch-ODC1 mRNA
AN3CA

Fig. S8. Knocking-down HOXB9 and ODC1 inhibit tumor growth in xenografts of HFD induced EC mice model (related to Figure 6)

(a) Ishikawa cells (stable transfection: sgNC/sg*HOXB9*-exon1/sg*ODC1*-exon4): ODC1/HOXB9 protein + grayscale quantification; right: *ODC1/HOXB9* mRNA. Data: mean ± SEM (n=3 independent experiments). Two-tailed One-way ANOVA for protein; Two-tailed Student's t-test for RNA. Same-experiment samples on different gels (ODC1, GAPDH; HOXB9), processed in parallel. (b) Subcutaneous sg model: mouse renal capsule plane MRI (fat content, yellow; High-fat diet/Low-fat diet [HFD/LFD]: n=3 each, after 2-month diet). (c) Subcutaneous sg model: mouse fat/lean content (HFD/LFD: n=6 each). Data: mean ± SEM. Two-tailed Student's t-test. (d) Subcutaneous sg model: mouse weight curves (n=6 per group). Data: mean ± SD. Two-tailed Two-way ANOVA. (e) Subcutaneous sg model: tumor growth curves (n=6 per group). Data: mean ± SD. Two-tailed One-way ANOVA. (f) Subcutaneous sg model: xenograft weights (experiment end, n=6 per group). Data: mean ± SD. Two-tailed One-way ANOVA. (g) Subcutaneous sg model: xenograft lysate ODC1/HOXB9 (WB, n=6 per group). Lower: grayscale. No tumor: WB signal=0. Data: mean ± SD. Two-tailed One-way ANOVA. Same-experiment samples on different gels (ODC1, GAPDH; HOXB9), processed in parallel. (h) Subcutaneous sg model: xenograft *ODC1/HOXB9* mRNA (n=6 per group). No tumor: mRNA=0. (i) AN3CA cells (stable transfection: shNC/sh*HOXB9*/sh*ODC1*): Upper: *ODC1/HOXB9* mRNA; Lower: protein + right: grayscale. Data: mean ± SEM (n=3 independent experiments). Two-tailed One-way ANOVA for protein; Two-tailed Student's t-test for RNA. Same-experiment samples on different gels (ODC1, GAPDH; HOXB9), processed in parallel. (j) Footpad lymph node metastasis (LNM) model: mouse serum BzCl-derivatized polyamines. Data: mean ± SD. Two-tailed One-way ANOVA. (k) Footpad LNM model: gross appearance of enlarged sub-iliac lymph nodes (2 groups). (l) Footpad LNM model: dissected sub-iliac lymph nodes (3 months post-inoculation, n=5 per group) + right: Lymph node area quantification. Data: mean ± SD. Two-tailed Student's t-test. (m) Footpad LNM model: lymph node IHC (anti-keratin/anti-CK18, n=5 per group). Scale bars: 100 µm (IHC), 20 µm (enlarged IHC).

*p < 0.05, **p < 0.01, ***p < 0.001, ****p < 0.0001. Multiple comparisons were corrected. Source data are provided as a Source Data file. Exact p-values are provided in the Source Data file.

Fig. S9

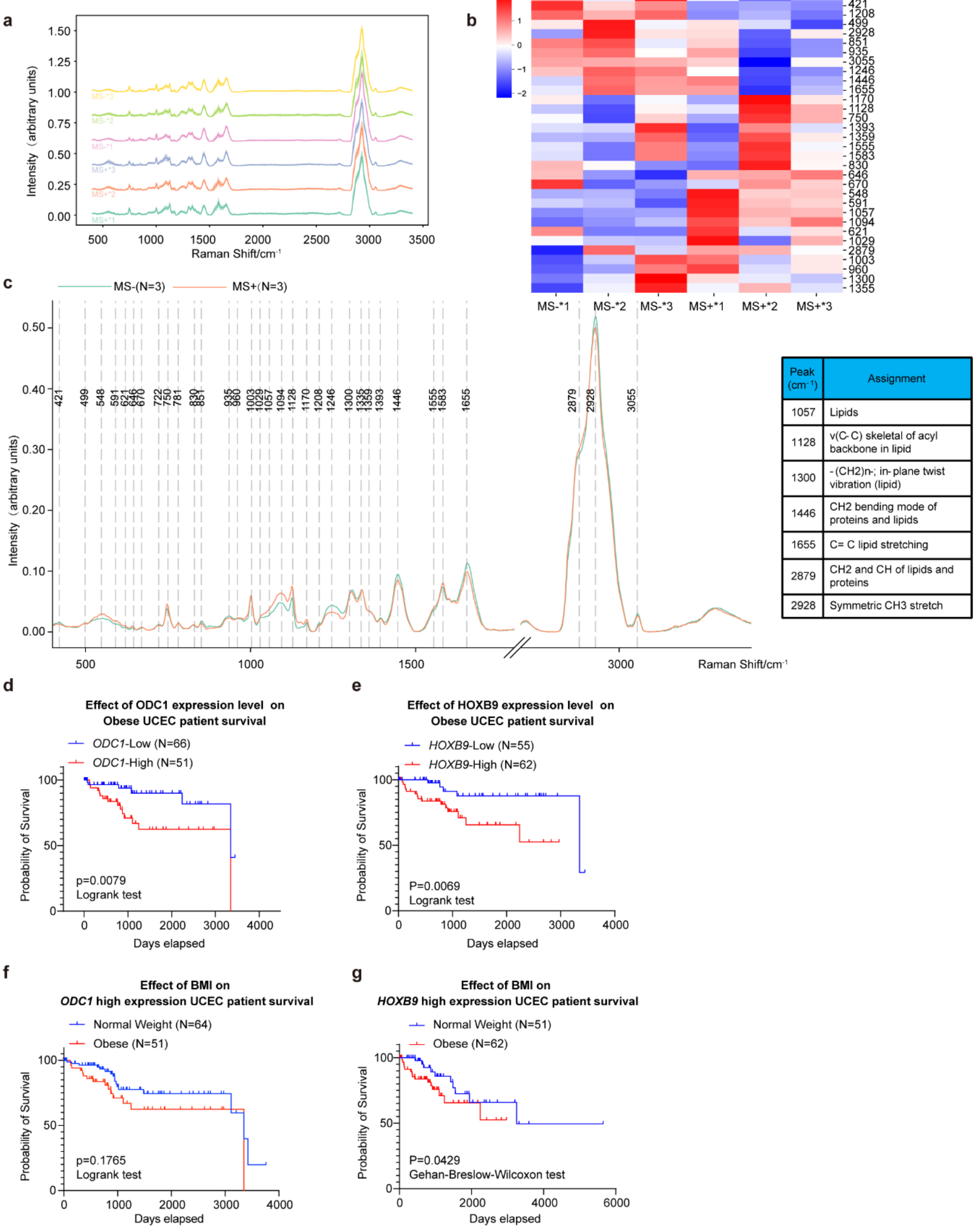

Peak (cm^{-1})	Assignment
1057	Lipids
1128	v(C-C) skeletal of acyl backbone in lipid
1300	-(CH2)n-; in-plane twist vibration (lipid)
1446	CH2 bending mode of proteins and lipids
1655	C= C lipid stretching
2879	CH2 and CH of lipids and proteins
2928	Symmetric CH3 stretch

Fig. S9. Raman spectroscopy revealed different lipid composition between EC samples with or without MS (related to Figure 7)

(a) Raman spectra of 6 samples. (b) Clustermap of Raman spectra of 6 samples (MS+: N=3; MS-: N=3). The color depth and tone of heat map indicate the correlation of data values. The tree

diagram of rows (Y-axis, Raman characteristic peak) and columns (X-axis, sample) shows the similarity between samples or features, and the closer the branches, the more similar they are. (c). Total Raman Shift with arrows indicating lipid peaks of the EC samples with or without MS (N=3 per group). (d), Survival analysis of The Cancer Genome Atlas-Uterine corpus endometrial carcinoma (TCGA-UCEC) patients with obesity (BMI ≥ 30 and BMI < 35) stratified by *ODC1* mRNA expression levels. p-value: Log-rank (Mantel-Cox) test. (e), Survival analysis of TCGA-UCEC patients with obesity (BMI ≥ 30 and BMI < 35) stratified by *HOXB9* mRNA expression levels. p-value: Log-rank (Mantel-Cox) test. (f), Survival analysis of different body weight groups in TCGA-UCEC with high *ODC1* mRNA expression patients. p-value: Log-rank (Mantel-Cox) test. (g), Survival analysis of different body weight groups in TCGA-UCEC patients with high *HOXB9* mRNA expression. Significant difference was detected by Gehan-Breslow-Wilcoxon test (p = 0.0429), indicating survival divergence primarily in the early follow-up period; Log-rank (Mantel-Cox) test showed no significance (p = 0.1283). Source data are provided as a Source Data file.

Fig. S10

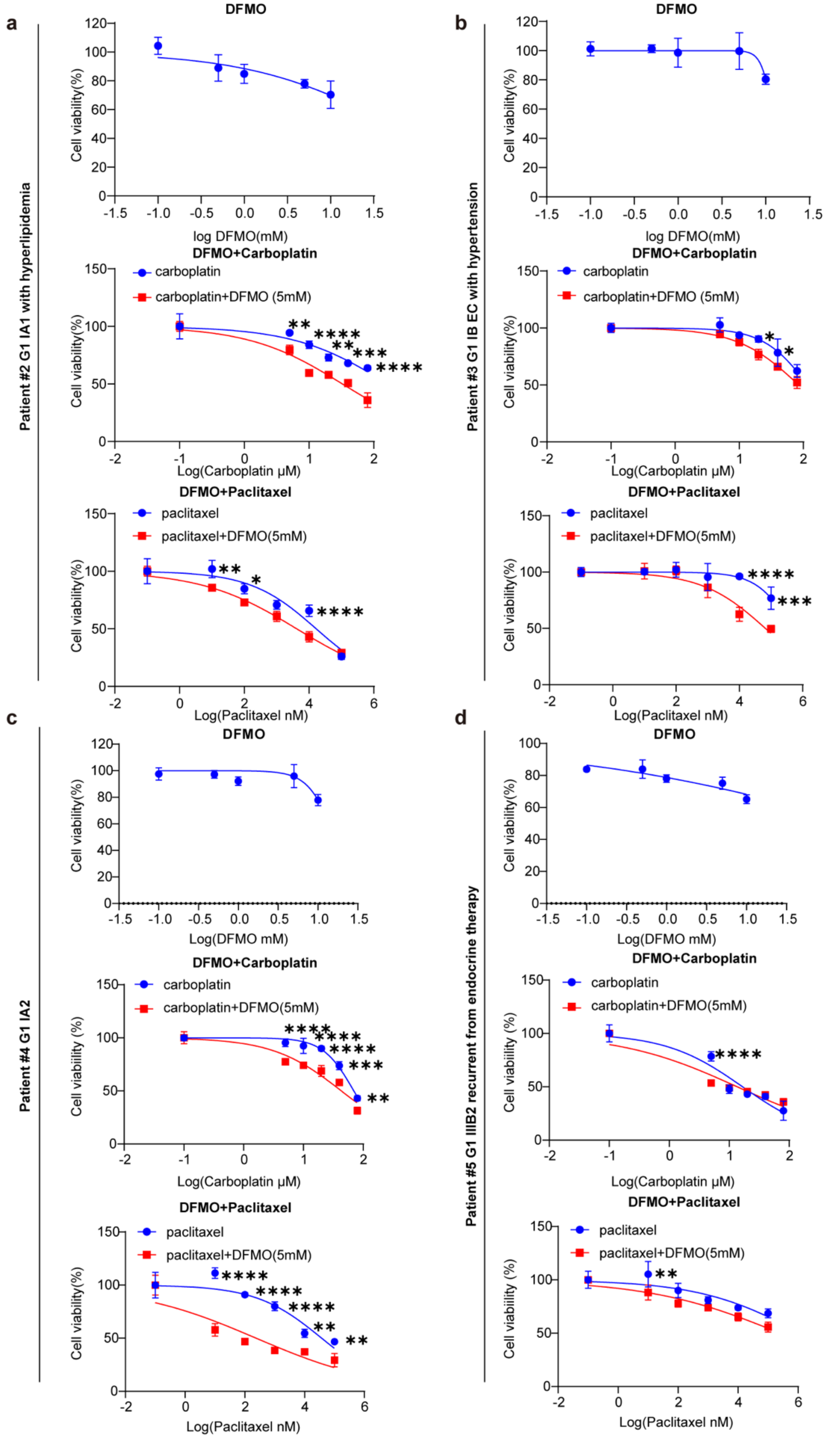
a
Patient #2 G1 IA1 with hyperlipidemia
DFMO
Cell viability(%)
log DFMO(mM)
DFMO+Carboplatin
carboplatin
carboplatin+DFMO (5mM)
Log(Carboplatin μM)
DFMO+Paclitaxel
paclitaxel
paclitaxel+DFMO(5mM)
Log(Paclitaxel nM)
b
Patient #3 G1 IB EC with hypertension
DFMO
log DFMO(mM)
DFMO+Carboplatin
Log(Carboplatin μM)
DFMO+Paclitaxel
Log(Paclitaxel nM)
c
Patient #4 G1 IA2
DFMO
Log(DFMO mM)
DFMO+Carboplatin
carboplatin+DFMO(5mM)
Log(Carboplatin μM)
DFMO+Paclitaxel
Log(Paclitaxel nM)
d
Patient #5 G1 IIIB2 recurrent from endocrine therapy
DFMO
Log(DFMO mM)
DFMO+Carboplatin
Log(Carboplatin μM)
DFMO+Paclitaxel
Cell viability (%)
Log(Paclitaxel nM)

Fig. S10. DFMO can improve the chemosensitivity of PTCs from chemotherapy-resistant EC patients (related to Figure 7).

(a) Patient-derived tumor cells (PTCs) from a stage IA1 Grade 1 EC patient with hyperlipidemia. Viability after 7-day treatment with varying concentrations of Eflornithine (DFMO), paclitaxel, carboplatin, or DFMO (5mM) co-administration. Data: mean ± SD (n = 3 replicate wells) *p < 0.05, **p < 0.01, ***p < 0.001, ****p < 0.0001 Two-way ANOVA. (b) PTCs from a stage IB Grade 1 EC patient with hypertension. Viability after 7-day treatment with varying concentrations of DFMO, paclitaxel, carboplatin, or DFMO (5mM) co-administration. Data: mean ± SD (n = 3 replicate wells) *p < 0.05, **p < 0.01, ***p < 0.001, ****p < 0.0001 Two-way ANOVA. (c) PTCs from a stage IA2 Grade 1 EC patient. Viability after 7-day treatment with varying concentrations of DFMO, paclitaxel, carboplatin, or DFMO (5mM) co-administration. Data: mean ± SD (n = 3 replicate wells) *p < 0.05, **p < 0.01, ***p < 0.001, ****p < 0.0001 Two-way ANOVA. (d) PTCs from a stage IIIB2 Grade 1 EC patient with recurrence after endocrine treatment. Viability after 7-day treatment with varying concentrations of DFMO, paclitaxel, carboplatin, or DFMO (5mM) co-administration. Data: mean ± SD (n = 3 replicate wells) *p < 0.05, **p < 0.01, ***p < 0.001, ****p < 0.0001 Two-way ANOVA. Source data are provided as a Source Data file. Exact p-values are provided in the Source Data file.

Fig. S11

a

b

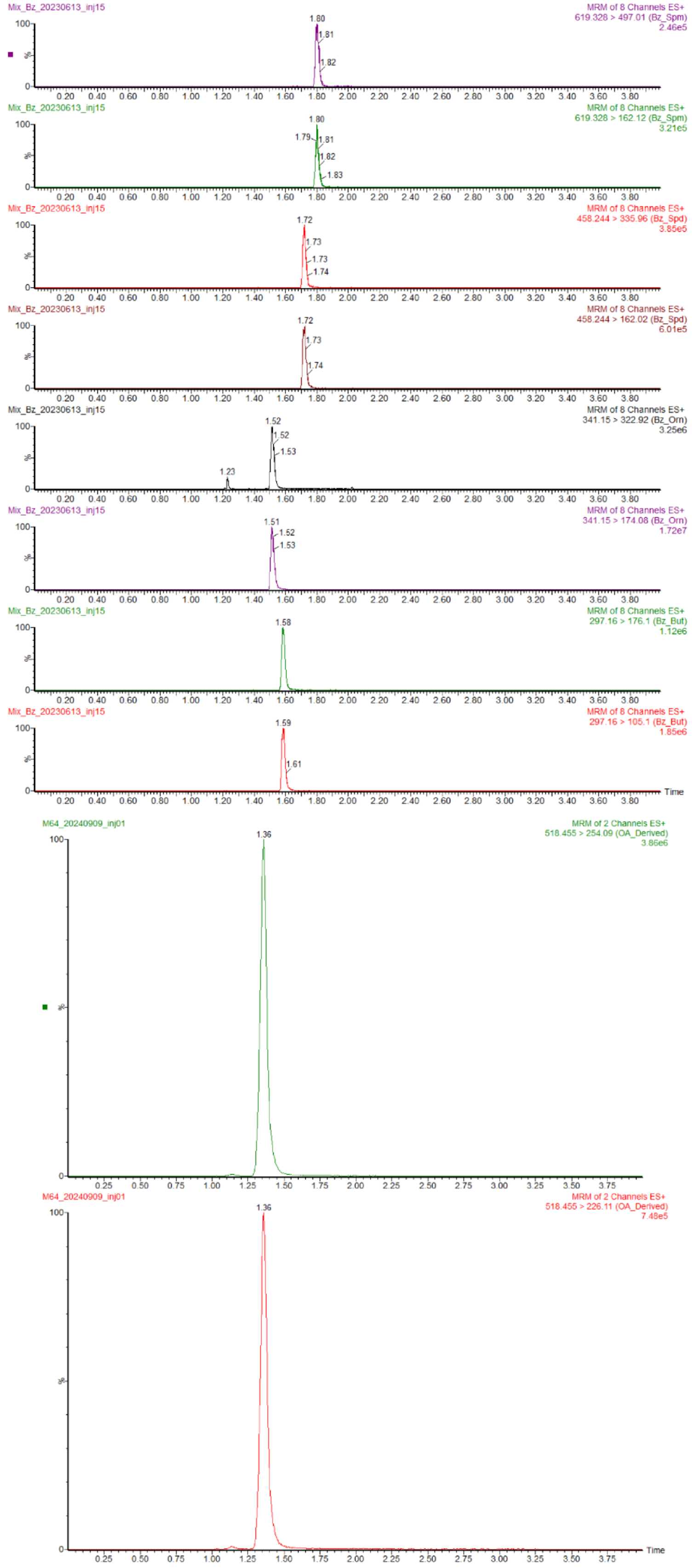

Mix_Bz_20230613_inj15
MRM of 8 Channels ES+
619.328 > 497.01 (Bz_Spm)
2.46e5
619.328 > 162.12 (Bz_Spm)
3.21e5
458.244 > 335.96 (Bz_Spd)
3.85e5
458.244 > 162.02 (Bz_Spd)
6.01e5
341.15 > 322.92 (Bz_Orn)
3.25e6
341.15 > 174.08 (Bz_Orn)
1.72e7
297.16 > 176.1 (Bz_But)
1.12e6
297.16 > 105.1 (Bz_But)
1.85e6
Time
M64_20240909_inj01
MRM of 2 Channels ES+
518.455 > 254.09 (OA_Derived)
3.86e6
518.455 > 226.11 (OA_Derived)
7.48e5
Time

Fig. S11. Ion pair information of BzCl derived polyamines and T3 derived OA in LC-MS (related to Method)

(a). Ion pair information of Benzoyl chloride (Bz/BzCl) derived polyamines (spermine [Spm], spermidine [Spd], putrescine [But], ornithine [Orn]) in LC-MS. (b). Ion pair information of 2,4-bis(diethylamino)-6-hydrazino-1,3,5-triazine (T3) derived Oleic acid (OA) in LC-MS.

Table S1. The clinical characteristics of postmenopausal endometrioid EC patients with or without MS in the identification cohort.

EC without MS				EC with MS					
Variables		N	Percent	Variables		N	Percent	χ²/t test	P
Total number		32		Total number		30			
Age (years)				Age (years)				18.4	<0.0001
	<50	2	6.25%						
	≥50, <60	22	68.75%		<60	7	23.33%		
	≥60, <70	6	18.75%		≥60, <70	18	60.00%		
	≥70	1	3.13%		≥70	5	16.67%		
	N/A	1	3.13%						
Menopause				Menopause					
	No	0	0.00%		No	0	0.00%		
	Yes	32	100.00%		Yes	30	100.00%		
BMI				BMI				49.2[#]	<0.0001[#]
	<24	24	75.00%		≥24, <28	13	43.33%		
	≥24	8	25.00%		≥28, <32	11	36.67%		
					≥32	6	20.00%		
Hypertension				Hypertension				58.06[*]	<0.0001
	No	32	100.00%		No	0	0.00%		
	Yes	0	0.00%		Yes	30	100.00%		
Diabetes				Diabetes			0.00%	58.06[*]	<0.0001
	No	32	100.00%		No	0	0.00%		
	Yes	0	0.00%		Yes	30	100.00%		
				Including metformin		13	43.3%		
				Including insulin		8	26.7%		
				Including other types		11	36.7%		
				Dietary restriction or no treatment		6	20%		
TG (mmol/L)				TG (mmol/L)					
	Mean+SD	1.32+0.57			Mean+SD	2.31+1.48		-3.32[a]	0.002
HDL (mmol/L)				HDL (mmol/L)					

	Mean+SD	1.21+0.51			Mean+SD	1.05+0.23		0.57[a]	0.122
Grade				Grade				3.57	0.168
	G1	7	21.88%		G1	13	43.33%		
	G2	14	43.75%		G2	11	36.67%		
	G3	11	34.38%		G3	6	20.00%		
Stage				Stage				8.55	0.287
	IA	24	75.00%		IA	21	70.00%		
	IB	5	15.63%		IB	6	20.00%		
	IIB	1	3.13%		IIIA	1	3.33%		
	IIIC	1	3.13%		IIIC	2	6.66%		
	IVB	1	3.13%						
LVSI				LVSI				0.18	0.667
	Negative	23	71.88%		Negative	23	76.67%		
	Positive	9	28.13%		Positive	7	23.33%		
LNM				LNM				<0.0001*	1
	Negative	29	90.63%		Negative	28	93.33%		
	Positive	3	9.38%		Positive	2	6.67%		
MI				MI				0.555	0.456
	Superficial	26	81.25%		No	22			
	Deep	6	18.75%		Yes	8			
Cervical Involvement				Cervical Involvement				1.27	0.26
	No	29	90.63%		No	30	100.00%		
	Yes	3	9.38%		Yes	0	0.00%		
Ovary Involvement				Ovary Involvement			0.00%		
	No	30	93.75%		No	28	93.33%	<0.0001*	1
	Yes	2	6.25%		Yes	2	6.67%		
Tumor Size (cm^3)				Tumor Size (cm^3)				4.79	0.091
	<2	10	31.25%		<2	12	40.00%		
	>2	15	46.88%		>2	6	20.00%		

	>4	7	21.88%		>4	11	36.67%		
					N/A	1	3.33%		
Endocrine Therapy				Endocrine Therapy			0.00%	<0.0001*	1
	No	30	93.75%		No	26	86.67%		
	Yes	2	6.25%		Yes	1	3.33%		
					N/A	3	10.00%		
Radio Therapy				Radio Therapy				2.536	0.111
	No	21	65.6%		No	25	74.2%		
	Yes	11	34.3%		Yes	5	25.8%		
Chemotherapy				Chemotherapy				0.969	0.325
	No	22	68.8%		No	17	56.7%		
	Yes	10	31.3%		Yes	13	43.3%		
Recurrence				Recurrence				0.008*	0.928
	Yes	3	9.4%		Yes	4[b]	13.3%		
	No	29	90.6%		No	26	86.7%		
Death				Death				0.341*	0.559
	No	31	96.9 %		No	27	90%		
	Yes	1	3.1%		Yes	3	10%		
DFS (days)				DFS (days)				-0.642[a]	0.524
	Mean+SD	3197.12+622.85			Mean+SD	4271.19+8512.9			
OS (days)				OS (days)				-0.648[a]	0.520
	Mean+SD	3334.42+407.36			Mean+SD	4410.77+8641.6			

EC, Endometrial cancer. MS, Metabolic syndrome. BMI, body mass index. TG, Total Triglyceride. HDL, High Density Lipoprotein. LVSI, lymph vascular space invasion. LNM, lymph node metastases. MI, Myometrial invasion. DFS, disease free survival. [#] Fisher Precise significance. [*]Continuity correction. [a] student's t-test. [b]Developed hyperlipidemia during follow-up. The chi-square/t-test is two-tailed.

Table S2. Important features identified by PLS-DA.

Compounds	Log2 FoldChange	p.value	VIP
PC(p36:4)	1.060462488	3.29E-12	3.159361
Nicotine	1.808385051	6.92E-12	3.128184
Spermine	1.129678673	4.50E-11	3.044617
Glutathione	1.527821022	3.67E-09	2.815611
PC(36:4)	0.6903	6.74E-07	2.672555
LysoPC(18:0)	-1.247319906	0.000102	2.647619
LysoPC(20:0)	-0.771125911	9.04E-08	2.613109
LPC(19:0)	-0.640771221	1.33E-07	2.585969
N-acetylputrescine	0.42767	6.45E-05	2.561826
PC(40：2)	0.40396	3.93E-10	2.518572
L-Pipecolic acid-2	-1.227320418	2.36E-06	2.364848
Glucosamine	0.60814687	5.22E-06	2.296517
D-Mannosamine	0.60814687	5.22E-06	2.296517
PC(34:2)	0.808508617	1.40E-05	2.206158
PC(p16:0/22:5)/PC(p18:1/20:4)	0.37319	0.000212	2.168593
LPE(18:0e)	-0.734923223	2.18E-05	2.163588
LPI(16:0)	-0.73626846	5.04E-05	2.078839
METHACHOLINE-like	0.685984987	0.000107	1.998424
Metformin	7.344739746	0.000395	1.845878
Biliverdin	-0.706723697	0.009307	1.387208
N-acetyl-L-tyrosine ethyl ester	0.951513196	0.023041	1.220381
Trimethylamine N-oxide(TMAO)	0.671474547	0.023678	1.215015
Arg-Val	0.757450016	0.042358	1.094763
Val-Arg	0.757450016	0.042358	1.094763
Hydroxyhexanoycarnitine	0.638398262	0.042933	1.091833

Table S3. The clinical characteristics of postmenopausal endometrioid EC patients in the validation cohort.

Variables		Number	Percent
Total number		156	
Dyslipidemia			
	No	63	40.38%
	Yes	92	58.97%
	N/A	1	0.64%
Diabetes			
	No	97	62.18%
	Yes	58	37.18%
	N/A	1	0.64%
Hypertension			
	No	112	71.79%
	Yes	44	28.21%
Recurrence or Metastasis			
	No	138	88.46%
	Yes	18	11.54%
Grade			
	1	67	42.95%
	2	58	37.18%
	3	31	19.87%
Stage			
	I	128	82.05%
	II	6	3.85%
	III	10	6.41%
	IV	9	5.77%
	N/A	3	1.92%
Myometrial Invasion			
	Superficial	105	67.31%
	Deep	45	28.85%
	N/A	6	3.85%
LNM			
	Negative	139	89.10%
	Positive	10	6.41%
	N/A	7	4.49%
LVSI			
	Negative	109	69.87%
	Positive	39	25.00%
	N/A	8	5.13%
Cervical Involvement			
	Negative	139	89.10%
	Positive	10	6.41%
	N/A	7	4.49%
Ovary Involvement			

	Negative	144	92.31%
	Positive	5	3.21%
	N/A	7	4.49%

LVSI, lymph vascular space invasion. LNM, lymph node metastases.

Table S4. The clinical characteristics of postmenopausal endometrioid EC patients with or without MS in the validation cohort.

EC without MS				EC with MS					
Variables		N	Percent	Variables		N	Percent	χ^2	P
Total number		28		Total number		22			
Menopause				Menopause					
	No	0	0.00%		No	0	0.00%		
	Yes	28	100.00%		Yes	22	100.00%		
Hypertension				Hypertension				50	<0.0001
	No	28	100.00%		No	0	0.00%		
	Yes	0	0.00%		Yes	22	100.00%		
Diabetes				Diabetes			0.00%	50	<0.0001
	No	28	100.00%		No	0	0.00%		
	Yes	0	0.00%		Yes	22	100.00%		
				Including metformin		12	54.5%		
				Including insulin		2	9.1%		
				Including other types		3	13.6%		
				Dietary restriction or no treatment		8	36.4%		
Hyperlipidemia				Hyperlipidemia				50	<0.0001
	No	28			No	0			
	Yes	0			Yes	22			
Grade				Grade				2.09	0.654[b]
	G1	11	39.30%		G1	7	31.80%		
	G2	12	42.90%		G2	8	36.40%		
	G3	5	17.90%		G3	6	27.30%		
Stage				Stage				2.591	0.621[a]
	I	25	89.30%		I	18	81.80%		
	II	1	3.60%		II	0	0.00%		
	III-IV	2	7.10%		III-IV	4	18.10%		
LVSI				LVSI				0.314	0.75[b]
	Negative	20	71.43%		Negative	14	63.64%		
	Positive	7	25.00%		Positive	7	31.82%		
	NA	1	3.57%			1	4.55%		
LNM				LNM				3.922	0.084[a]
	Negative	27	96.43%		Negative	19	86.36%		
	Positive	0	0.00%		Positive	3	13.64%		
	NA	1	3.57%						
MI				MI				2.975	0.127[b]
	Superficial	21	75.00%		No	12	54.55%		

	Deep	6	21.43%		Yes	10	45.45%		
	NA	1	3.57%						
Cervical Involvement				Cervical Involvement				0.000[*]	1.00[#]
	No	26	92.86%		No	20	90.91%		
	Yes	1	3.57%		Yes	1	4.55%		
	NA	1	3.57%		NA	1	4.55%		
Ovary Involvement				Ovary Involvement			0.00%	1.253	0.449[a]
	No	27	96.43%		No	21	95.45%		
	Yes	0			Yes	1	4.55%		
	NA	1	3.57%						
recurrence or metastasis				recurrence or metastasis				0.568[*]	0.451[#]
	No	26	92.86%		No	18	81.82%		
	Yes	2	7.14%		Yes	4	18.18%		

EC, Endometrial cancer. MS, Metabolic syndrome. LVSI, lymph vascular space invasion. LNM, lymph node metastases. MI, Myometrial invasion. [*]Continuity correction. [#]Asymptotic significance. [a] Fisher Precise significance. [b] Precise significance. The chi-square test is two-tailed.

Table S5. The clinical characteristics of EC Patients with Pathologic Presence of LVSI/LNM Between 2014-2020

Variables		Number	Percent
Total number		17	
Age (years)			
	Mean±SD	62±8	
BMI			
	<24	2	11.76%
	⩾24, <28	7	41.18%
	⩾28, <32	7	41.18%
	⩾32	1	5.88%
Hypertension			
	No	7	41.18%
	Yes	10	58.85%
Diabetes			
	No	10	58.82%
	Yes	7	41.18%
Stage			
	Stage I	11	64.71%
	Stage II-IV	6	35.29%
Tumor grade			
	G1	7	41.18%
	G2	6	35.29%
	G3	4	23.53%
LVSI			
	Negative	1	5.88%
	Positive	16	94.12%
LNM			
	Negative	12	70.59%
	Positive	5	29.41%

BMI, body mass index. LVSI, lymph vascular space invasion. LNM, lymph node metastases.

Table S6. The clinical characteristics of EC TMA.

Patient characteristics		N	Percent
Number			
	Total	135	
	Cancer	118	87.41%
	Para-cancer	17	12.559%
Age (years)			
	Mean±SD	54±8	
MI	None	2	1.69%
	Superficial	70	59.32%
	Deep	35	29.66%
	NA	11	9.32%
Grade			
	G1	34	28.81%
	G2	57	48.31%
	G3	25	21.19%
LNM			
	Negative	102	86.44%
	Positive	5	4.24%
	NA	11	9.32%
Stage			
	IA	73	61.86%
	IB	22	18.64%
	II	11	9.32%
	IIIA	4	3.39%
	IIIB	1	0.85%
	IIIC	6	5.08%
	IV	1	0.85%
Diabetes			
	No	99	83.90%
	Yes	18	15.25%
Survival			
	Yes	98	83.05%
	No	20	16.95%
PFS (days)	Mean±SD		88.38+22.61

EC, Endometrial cancer. TMA, Tissue Microarray. MI, Myometrial invasion. LNM, lymph node metastases. PFS, progression free survival.

Table S7. The clinical characteristics of patients' sample received Raman Spectrophy.

	MS-	Number	Percent	MS+	Number	Percent	χ^2/t test	P
Total		3			3			
Age (years)	Mean±SD	64±11		Mean±SD	68±2		-0.62*	0.57
grade	G1	1	33.33%	G2	3	100.00%	3	0.22
	G2	1	33.33%					
	G3	1	33.33%					
Stage	IA	1	33.33%	IA	1	33.33%	NA	1[a]
	IB	1	33.33%	IB	2	66.67%		
	IIC	1	33.33%					
MI	Yes	2	66.67%	Yes	2	66.67%	0#	1
	No	1	33.33%	No	1	33.33%		
LVSI	Yes	2	66.67%	Yes	1	33.33%	0#	1
	No	1	33.33%	No	2	66.67%		
LNM	Yes	0	0.00%	Yes	0	0.00%	NA	NA
	No	3	100.00%	No	3	100.00%		
Diabetes	Yes	0	0.00%	Yes	3	100.00%	NA	0.1 [a]
	No	3	100.00%	No	0	0.00%		
Obesity	Yes	0	0.00%	Yes	3	100.00%	NA	0.1 [a]
	No	3	100.00%	No	0	0.00%		
Hypertension	Yes	0	0.00%	Yes	2	66.67%	NA	0.4 [a]
	No	3	100.00%	No	1	33.33%		

MI, Myometrial invasion. LNM, lymph node metastases. LVSI, lymph vascular space invasion. *t-test. #Continuity correction. [a] Fisher Precise significance. The chi-square/t-test is two-tailed.

Table S8. The clinical characteristics of PTCs received drug resistance experiments.

Patient characteristics		N	Percent
Number		5	
Age (years)			
	Mean±SD	42±10	
MI			
	None	2	40.00%
	Superficial	2	40.00%
	Deep	1	20.00%
Grade			
	G1	4	80.00%
	G2	1	20.00%
LNM			
	Negative	5	100.00%
	Positive	0	0.00%
LVSI			
	Negative	5	100.00%
	Positive	0	0.00%
Stage			
	IA	3	60.00%
	IB	1	20.00%
	IIIB	1	20.00%
hyperlipidemia			
	Negative	3	60.00%
	Positive	2	40.00%
Diabetes			
	Negative	5	100.00%
	Positive	0	0.00%
Hypertension			
	Negative	4	80.00%
	Positive	1	20.00%
Cervical involvement			
	Negative	5	100.00%
	Positive	0	0.00%
Parametrial infiltration			
	Negative	4	80.00%
	Positive	1	20.00%

MI, Myometrial invasion. LNM, lymph node metastases. LVSI, lymph vascular space invasion.

Table S9. The antibodies.

Antibody	Supplier name	Catalog number	Clone name	Lot number
ODC1 Rabbit mAb	Abclonal	A3898	ARC0863	4000000863
ODC1 Rabbit pAb	Proteintech	28728-1-AP-50UL	Polyclonal	00118403
ODC1 Mouse mAb	Santa Cruz Biotechnology	sc-390366	G10	#C0420
ODC1 Rabbit mAb	Abcam	ab270268	ODC1/2878R	1081038-2
OAZ1 Rabbit pAb	Abclonal	A7444	Polyclonal	0037840102
SRM Rabbit pAb	Proteintech	19858-1-AP	Polyclonal	00085878
SMS Rabbit pAb	Proteintech	15979-1-AP-50UL	Polyclonal	00007226
SMS Mouse mAb	Proteintech	68040-1-Ig-50UL	1G8E9	10024538
HOXB9(H-8) Mouse mAb	Santa Cruz Biotechnology	sc-398500	H-8	#K1921
HOXB9 Rabbit mAb	Abcam	ab133701	EPR6950	GR3452370-1
Praja2 (PJA2) Rabbit pAb	Bethyl Laboratories	A302-992A-T	Polyclonal	#1
SREBF1 Mouse mAb	Proteintech	66875-1-Ig	1B6G5	10020404
ZEB1 (D80D3) Rabbit mAb	Cell Signaling Technology	#3396	D80D3	10
Vimentin Rabbit mAb	Abcam	ab92547	EPR3776	GR3258719-43
EZH2 Rabbit mAb	Cell Signaling Technology	#5246	D2C9	10
Flag Rabbit mAb	Abclonal	AE063	ARC5111-02	3522060804
anti HA-Tag Mouse mAb	Abclonal	AE008	AMC0503	6100005027
GFP Rabbit mAb	Abclonal	AE078	ARC50809	356122205
β-Actin Mouse mAb	Proteintech	66009-1-Ig	2D4H5	10024215
GAPDH Recombinant mAb	Proteintech	81640-5-RR	1H18	23011777
Lamin B1 Rabbit mAb	Cell Signaling Technology	#13435	D9V6H	10
Ki67 Mouse mAb	Cell Signaling Technology	#9449	8D5	10
Pan-keratin Rabbit pAb	Proteintech	26411-1-AP	Polyclonal	00114730
Cytokeratin 18 Mouse mAb	Santa Cruz Biotechnology	sc-6259	DC-10	B1523
HRP-conjugated Mouse Anti-Rabbit IgG Light Chain	Abclonal	AS061	AMC0531	9300061003
Protein G PLUS-Agarose	Santa Cruz Biotechnology	sc-2002	#G3120	#C0223
Anti-DYKDDDDK (Flag) Affinity Gel	Yeason	20584ES25	1A3	A7407130
Protein A-Agarose	Santa Cruz Biotechnology	sc-2001	#J1222	#J2521
Rabbit Control IgG	Abclonal	AC005)	AC005	3411056789
Mouse Control IgG	Abclonal	AC011	AC011	3600004335

Table S10. The gradient elution program for mobile phases A and B in the positive ion mode of untargeted metabolomics.

Time(min)	A (%)	B (%)
0	99	1
2	99	1
3	95	5
11	40	60
14	40	60
15	1	99
17	1	99
17.1	99	1
20	99	1

A (%) and B (%) represent the volume percentages of Mobile Phase A and Mobile Phase B in the total mobile phase at specific times.

Table S11. The gradient elution program for mobile phases A and B in the negative ion mode of untargeted metabolomics.

Time (min)	A (%)	B (%)
0	99	1
3	99	1
12	1	99
17	1	99
17.1	99	1
20	99	1

A (%) and B (%) represent the volume percentages of Mobile Phase A and Mobile Phase B in the total mobile phase at specific times.

Table S12. The ion pair information of BzCl-polyamines in LC-MS.

ID	Chemical compound	Ion pair
1	Bz_Orn	341.15>174.08
		341.15>322.92
2	Bz_Put	297.16> 105.1
		297.16> 176.1
3	Bz_Spd	458.244 > 162.02
		458.244>335.96
4	Bz_Spm	619.328>162.12
		619.328 >497.01
