# Supplementary material for: Metabolic syndrome promotes endometrial cancer by Oleic acid-mediated polyamine accumulation
Source: Nat Commun. 2025 Dec 16;17:388. doi: 10.1038/s41467-025-67083-y (PMC12796254; doi:10.1038/s41467-025-67083-y)
Supplement: Supplementary file 2 — Reporting Summary [file 41467_2025_67083_MOESM2_ESM.pdf]

nature portfolio

Corresponding author(s): Jianliu Wang and Jun Zhan

Last updated by author(s): Oct 12, 2025

Reporting Summary

Nature Portfolio wishes to improve the reproducibility of the work that we publish. This form provides structure for consistency and transparency in reporting. For further information on Nature Portfolio policies, see our Editorial Policies and the Editorial Policy Checklist.

Statistics

For all statistical analyses, confirm that the following items are present in the figure legend, table legend, main text, or Methods section.

n/a	Confirmed	
☐	☒	The exact sample size (n) for each experimental group/condition, given as a discrete number and unit of measurement
☐	☒	A statement on whether measurements were taken from distinct samples or whether the same sample was measured repeatedly
☐	☒	The statistical test(s) used AND whether they are one- or two-sided<br>*Only common tests should be described solely by name; describe more complex techniques in the Methods section.*
☐	☒	A description of all covariates tested
☐	☒	A description of any assumptions or corrections, such as tests of normality and adjustment for multiple comparisons
☐	☒	A full description of the statistical parameters including central tendency (e.g. means) or other basic estimates (e.g. regression coefficient) AND variation (e.g. standard deviation) or associated estimates of uncertainty (e.g. confidence intervals)
☐	☒	For null hypothesis testing, the test statistic (e.g. F, t, r) with confidence intervals, effect sizes, degrees of freedom and P value noted<br>*Give P values as exact values whenever suitable.*
☒	☐	For Bayesian analysis, information on the choice of priors and Markov chain Monte Carlo settings
☒	☐	For hierarchical and complex designs, identification of the appropriate level for tests and full reporting of outcomes
☐	☒	Estimates of effect sizes (e.g. Cohen's d, Pearson's r), indicating how they were calculated

Our web collection on statistics for biologists contains articles on many of the points above.

Software and code

Policy information about availability of computer code

Data collection	Microsoft Excel 16.98.
Data analysis	Microsoft Excel 16.98; Graphpad Prism9.0; Image J; SPSS 25.0.

For manuscripts utilizing custom algorithms or software that are central to the research but not yet described in published literature, software must be made available to editors and reviewers. We strongly encourage code deposition in a community repository (e.g. GitHub). See the Nature Portfolio guidelines for submitting code & software for further information.

Data

Policy information about availability of data

All manuscripts must include a data availability statement. This statement should provide the following information, where applicable:

- Accession codes, unique identifiers, or web links for publicly available datasets
- A description of any restrictions on data availability
- For clinical datasets or third party data, please ensure that the statement adheres to our policy

All data supporting the findings of this study are available within the paper and its Supplementary Information. Source data are provided with this paper.

Research involving human participants, their data, or biological material

Policy information about studies with human participants or human data. See also policy information about sex, gender (identity/presentation), and sexual orientation and race, ethnicity and racism.

Reporting on sex and gender	The participants in this study were exclusively female patients with endometrial cancer. All analyses and interpretations of the study results are based on this female cohort, considering the specific physiological and clinical characteristics associated with female patients in the context of endometrial cancer.
Reporting on race, ethnicity, or other socially relevant groupings	No specific data on race or ethnicity of the participants were collected or reported in this study.
Population characteristics	All detailed population characteristics of the participants, including demographic information, clinical parameters, and pathological features, are comprehensively presented in the supplementary tables. Corresponding statistical tests have also been conducted to determine whether these population characteristics act as covariates influencing our conclusions.

Recruitment

Patient Recruitment

This study was an observational study and did not involve random assignment (a design feature exclusive to interventional studies, where participants are randomly allocated to intervention or control groups to balance confounding factors). However, random selection was adopted for specific cohorts to reduce selection bias.

This study was approved by the Ethics Committee of Peking University People's Hospital (approval number: 2022PHB372-001), and all participants provided written informed consent prior to enrollment, in full compliance with ethical guidelines.

1. Recruitment Process

All participants were recruited from the Gynecologic Oncology Outpatient Clinic and Inpatient Department of Peking University People's Hospital. Recruitment was conducted via the following steps:

Potential candidates were initially screened from the hospital’s electronic medical record (EMR) system based on the primary diagnosis of postmenopausal endometrial cancer.

The research team then verified key clinical and pathological information (e.g., surgical records, pathological reports, laboratory test results, and menopausal status documentation) to confirm preliminary eligibility.

Eligible patients were invited to participate in person, where the study purpose, procedures, potential risks, and data usage were explained in detail. Enrollment was finalized only after patients signed the written informed consent form.

Recruitment timelines and cohort-specific details are as follows:

Untargeted serum metabolomics cohort: 62 postmenopausal endometrial cancer patients (recruited 2012–2018), including 30 with metabolic syndrome (MS) and 32 without. MS was diagnosed per the 2013 criteria of the Diabetes Branch of the Chinese Medical Association (≥3 of: female waist circumference >85 cm, hyperglycemia, hypertension, fasting triglycerides ≥1.70 mmol/L, or fasting HDL <1.04 mmol/L).

Targeted polyamine metabolomics cohort: 156 postmenopausal patients with endometrioid endometrial cancer (recruited 2021–2022).

IHC cohort: 17 postmenopausal endometrial cancer patients with pathological evidence of lymphovascular space invasion (LVSI) or lymph node metastasis (LNM; recruited 2014–2020).

Additional experimental cohorts: 6 frozen endometrial cancer tissue samples (for Raman spectroscopy) and 7 patients (for patient-derived tumor cell [PTC]-based drug sensitivity tests) were randomly selected from postmenopausal endometrial cancer patients admitted to the hospital between June 2024 and June 2025. This random selection was intended to reduce selection bias by avoiding subjective preference in sample picking (not to achieve random group assignment).

2. Inclusion and Exclusion Criteria

Inclusion criteria: Confirmed diagnosis of endometrial cancer via pathological examination; documented postmenopausal status; and complete willingness to provide clinical data and biological samples.Exclusion criteria: Incomplete clinical/pathological data; concurrent severe systemic diseases; prior participation in other interventional studies that may interfere with outcomes; history of other malignant tumors; failure to meet postmenopausal status criteria.

These criteria were strictly applied to ensure data integrity and cohort homogeneity across analyses. Clinicopathological details of all cohorts are provided in Supplementary Tables 1, 3–8.

3. Potential Biases and Their Impact on Results

3.1 Selection Bias

Single-center recruitment: All participants were sourced from a single tertiary hospital (Peking University People's Hospital), which primarily serves patients with relatively complex or severe conditions (compared to primary/secondary hospitals) and may have a higher proportion of patients with access to tertiary care. This may limit the generalizability of results to patients treated in non-tertiary institutions or other geographic regions.

Retrospective cohort limitations: The untargeted metabolomics (2012–2018) and IHC (2014–2020) cohorts relied on retrospective EMR data, which may have introduced selection bias due to variations in historical documentation practices.

3.2 Self-Selection Bias

Eligible patients could decline participation due to time constraints, concerns about sample collection, or lack of interest. This may have led to overrepresentation of patients with higher health literacy or compliance, potentially skewing results.

3.3 Small-Sample Bias

The IHC (n=17), Raman spectroscopy (n=6), and PTC drug sensitivity (n=7) cohorts had small sample sizes. This increases the risk of random error and reduces statistical power, making it harder to detect true associations (e.g., between LVSI/LNM and

IHC markers) or generalize findings from these cohorts.

3.4 Mitigation Measures
To minimize biases, the study adopted:
Strict adherence to predefined inclusion/exclusion criteria (applied uniformly by two independent researchers);
Consistent diagnostic standards (e.g., MS diagnosis per national guidelines, pathological confirmation by two senior pathologists);
Transparent documentation of cohort characteristics (e.g., age, stage, MS status) in supplementary tables;
Random selection for small experimental cohorts (i.e., randomly picking samples from eligible cases, not random assignment to groups) to avoid subjective sampling bias.

Ethics oversight

All patients have signed informed consent forms. This study was approved by the Ethics Committee of Peking University People's Hospital (Ethics approval number: 2022PHB372-001).

Note that full information on the approval of the study protocol must also be provided in the manuscript.

Field-specific reporting

Please select the one below that is the best fit for your research. If you are not sure, read the appropriate sections before making your selection.

[x] Life sciences [ ] Behavioural & social sciences [ ] Ecological, evolutionary & environmental sciences

For a reference copy of the document with all sections, see nature.com/documents/nr-reporting-summary-flat.pdf

Life sciences study design

All studies must disclose on these points even when the disclosure is negative.

Sample size

The study set multiple sample sizes according to different detection purposes, all matching the research type and feasibility:
Untargeted metabolomics analysis: A total of 62 postmenopausal patients were included (30 with metabolic syndrome (MS) and 32 without MS). The sample size was based on the accumulation of clinical cases from 2012 to 2018, and strictly followed the CDS 2013 version of MS diagnostic criteria (all 4 indicators must be met), ensuring the comparability of baseline characteristics between groups and providing a basic data volume for metabolic difference analysis.
Targeted polyamine metabolomics analysis: 156 postmenopausal patients with endometrioid endometrial cancer (EC) were included. The relatively large sample size was suitable for targeted verification of metabolites, improving the statistical test power.
The sample sizes for IHC detection (17 EC patients with LVSI or LNM), Raman spectroscopy (6 cases), and drug sensitivity tests (7 cases) were relatively small, consistent with the characteristics of exploratory studies (such as preliminary analysis of pathological mechanisms and verification of new technologies). The sample sizes were determined by the availability of clinical pathological specimens and the complexity of detection.

Data exclusions

Baseline exclusion: Patients who did not meet the "postmenopausal" status or the CDS 2013 version of MS diagnostic criteria were excluded; EC patients were pathologically confirmed, and non-endometrioid cancer types were excluded.

Replication

Samples in each experimental group were derived from independent patients (e.g., 62 cases, 156 cases, with each case representing a distinct individual). This design covers biological diversity and enhances the generalizability of results.For metabolomics analyses (untargeted/ targeted), batch-specific quality control (QC) samples were included, and 10% of samples were retested to verify detection stability. For immunohistochemical (IHC) staining, double-blind reading was adopted (results independently interpreted by two pathologists) to reduce subjective bias. Cell and molecular biology experiments (e.g., functional assays, co-immunoprecipitation [Co-IP], Western blotting [WB]) were independently repeated at least 3 times, with each independent repetition including at least 2 technical replicates to ensure result consistency.
Replication Outcomes: All biological and technical replications were successful, with no experimental findings failing to be reproduced.

Randomization

This study was designed as an observational study, with an emphasis on randomness in sample selection to ensure objectivity:
Patients were included based on clinical diagnosis and treatment records, e.g., cases meeting the eligibility criteria during the time periods of 2012–2018 and 2021–2022. While random grouping (a design feature of interventional studies) was not employed, the randomness of sampling was detailed in the Methods section: samples were randomly selected from the database of eligible cases rather than being included selectively, aiming to minimize selection bias.

Blinding

Regarding blinding to group allocation: Investigators were blinded during both data collection and analysis. Specifically, during the process of collecting experimental data (e.g., recording IHC staining signals, measuring metabolomic indicators) and subsequent data analysis, the investigators were unaware of the specific group allocation of each sample (e.g., grouping based on clinical subtypes or inclusion time periods).

Reporting for specific materials, systems and methods

We require information from authors about some types of materials, experimental systems and methods used in many studies. Here, indicate whether each material, system or method listed is relevant to your study. If you are not sure if a list item applies to your research, read the appropriate section before selecting a response.

Materials & experimental systems

n/a	Involved in the study	
☐	☒	Antibodies
☐	☒	Eukaryotic cell lines
☒	☐	Palaeontology and archaeology
☐	☒	Animals and other organisms
☒	☐	Clinical data
☒	☐	Dual use research of concern
☒	☐	Plants

Methods

n/a	Involved in the study	
☒	☐	ChIP-seq
☒	☐	Flow cytometry
☒	☐	MRI-based neuroimaging

Antibodies

Antibodies used

Antibody, supplier name, catalog number, clone name, lot number, dilution
ODC1 Rabbit mAb, Abclonal, A3898, ARC0863, 4000000863, WB: 1:1000.
ODC1 Rabbit pAb, Proteintech ,28728-1-AP, Polyclonal, 00118403, WB: 1:1000, IHC: 1:500.
ODC1 Mouse mAb, Santa Cruz Biotechnology, sc-390366, G10, #C0420, immunoprecipitation: 1:150 (cell lysate).
ODC1 Rabbit mAb, Abcam, ab270268, ODC1/2878R, 1081038-2, WB: 1-2 µg/mL
OAZ1 Rabbit pAb, Abclonal, A7444, Polyclonal, 0037840102, WB: 1:500 - 1:2000.
SRM Rabbit pAb, Proteintech, 19858-1-AP, Polyclonal, 00085878, WB: 1:1000, IHC: 1:500.
SMS Rabbit pAb, Proteintech, 15979-1-AP-50UL, Polyclonal, 00007226, WB: 1:1000.
SMS Mouse mAb, Proteintech, 8040-1-Ig-50UL, 1G8E9, 10024538, IHC: 1:200.
HOXB9(H-8) Mouse mAb, Santa Cruz Biotechnology, sc-398500, H-8, #K1921, WB:
1:100, immunoprecipitation: 1:150 (cell lysate), immunofluorescence: 1:100, IHC: 1:200
HOXB9 Rabbit mAb, Abcam, ab133701, EPR6950, GR3452370-1, immunofluorescence: 1:200.
Praja2 (PJA2) Rabbit pAb, Bethyl Laboratories, A302-992A-T, Polyclonal, #1, WB: 1:1000, immunofluorescence: 1:200.
SREBF1 Mouse mAb, Proteintech, 66875-1-Ig, 1B6G5, 10020404, WB: 1:1000.
ZEB1 (D80D3) Rabbit mAb, Cell Signaling Technology, #3396, D80D3, 10, WB: 1:1000,
Ki-67 (8D5) Mouse mAb, Cell Signaling Technology, #9449, 8D5, 10, IHC: 1:3000.
Pan-keratin (pan-cytokeratin) Rabbit pAb, Proteintech, 26411-1-AP, Polyclonal, 00114730, IHC: 1:1500, IF-P: 1:500.
Cytokeratin 18 Mouse mAb, Santa Cruz Biotechnology, sc-6259, DC-10, B1523, IHC: 1:100.
Vimentin Rabbit mAb, Abcam, ab92547, EPR3776, GR3258719-43, IF: 2 µg/mL.
Ezh2 (D2C9) XP® Rabbit mAb, Cell Signaling Technology, #5246, D2C9, 10, WB: 1:1000.
Flag Rabbit mAb, Abclonal, AE063, ARC5111-02, 3522060804, WB: 1:1000.
anti HA-Tag Mouse mAb, Abclonal, AE008, AMC0503, 6100005027, WB: 1:1000.
GFP Rabbit mAb, Abclonal, AE078, ARC50809, 356122205, WB: 1:1000.
β-Actin Mouse mAb, Proteintech, 66009-1-Ig, 2D4H5, 10024215, WB: 1:1000.
GAPDH Recombinant mAb, Proteintech, 81640-5-RR, 1H18, 23011777, WB: 1:1000.
Lamin B1 Rabbit mAb, Cell Signaling Technology, #13435, D9V6H, 10, WB: 1:1000.
HRP-conjugated Mouse Anti-Rabbit IgG Light Chain, ABclonal, AS061, AMC0531, 9300061003, WB: 1:3000.
Protein G PLUS-Agarose, Santa Cruz Biotechnology, sc-2002, #G3120, #C0223, 30 µl per immunoprecipitation reaction.
Protein A-Agarose, Santa Cruz Biotechnology, sc-2001, #J1222, #J2521, 30 µl per immunoprecipitation reaction.
Anti-DYKDDDDK (Flag) Affinity Gel: Yeason, 20584ES25, 1A3, A7407130, 10 µl per immunoprecipitation reaction.
Rabbit Control IgG, ABclonal, AC005, 3411056789, 0.5ug for 300ug cell lysates.
Mouse Control IgG, Abclonal, AC011, 3600004335, 0.5ug for 300ug cell lysates.

Validation

ODC1 Rabbit mAb (A3898) Abclonal; https://abclonal.com.cn/catalog/A3898 ; Figure3h upper; Figure 3b,3c; Figure 3e; 5b, 5h,
ODC1 Rabbit pAb (28728-1-AP-50UL) Proteintech; https://www.ptgcn.com/products/ODC1-Antibody-28728-1-AP.htm ; Figure1h; Figure3h lower; Figure 3d upper, 3i, 4b, Figure S5c; Figure S7f;
ODC1 Mouse mAb (sc-390366) Santa Cruz Biotechnology; https://datasheets.scbt.com/sc-390366.pdf ; Figure1j; Figure 3l; Figure 3f, 3m;5e; 7a,
ODC1 Rabbit mAb (ab270268) Abcam; https://www.abcam.com/en-us/products/primary-antibodies/ornithine-decarboxylase-odc-antibody-odc1-2878r-ab270268 ; Figure2a,2g;Figure 2l; Figure 3d lower; Figure 3f, 3g, 3j, 3k, 4a; Figure S3c, S4a, S4f, S4h; S4i, S5a, S5b, S7d, S7e, S7i, S8a, S8g, S8i,
OAZ1 Rabbit pAb (A7444) Abclonal; https://abclonal.com.cn/catalog/A7444 ; Figure 3j, 7a, ; Figure S4e
SRM Rabbit pAb (19858-1-AP) Proteintech; https://www.ptgcn.com/products/SRM-Antibody-19858-1-AP.htm ; Figure1h;Figure1j
SMS Rabbit pAb (15979-1-AP-50UL) Proteintech; https://www.ptgcn.com/products/SMS-Antibody-15979-1-AP.htm ; Figure1h
SMS Mouse mAb (68040-1-Ig-50UL) Proteintech; https://www.ptgcn.com/products/SMS-Antibody-68040-1-Ig.htm ; Figure1j
HOXB9(H-8) Mouse mAb (sc-398500) Santa Cruz Biotechnology; https://www.scbt.com/zh/p/hoxb9-antibody-h-8 ; Figure 3c, 3d, 3e, 3f, 3g, 3i, 3k, 3l, 3m, 4a, 4b, 5a, 5f; 5h, 7a, Figure S4f, Figure S4h, S5a, S5b, S7d, S7e, S7f, S7i, S8a, S8g, S8i,
HOXB9 Rabbit mAb (ab133701) Abcam; https://www.abcam.cn/products/primary-antibodies/hoxb9-antibody-epr6950-ab133701.html ; Figure 5e
praja2 Rabbit pAb (A302-991A) Bethyl Laboratories; https://www.biomol.com/products/antibodies/primary-antibodies/general/anti-pja2-a302-991a ; Figure 5b, 7a; Figure S4i

SREBF1 Mouse mAb (66875-1-Ig)Proteintech; https://www.ptgcn.com/Products/SREBF1-Antibody-66875-1-Ig.htm; Figure 3k, Figure S4f,
ZEB1 (D80D3) Rabbit mAb (#3396) Cell Signaling Technology; https://media.cellsignal.com/pdf/3396.pdf; Figure 2l; Figure 4a;
Ki-67 (8D5) Mouse mAb(#9449), Cell Signaling Technology; https://www.cellsignal.com/products/primary-antibodies/ki-67-8d5-mouse-mab/9449； Figure S7f.
Pan-keratin Rabbit pAb (26411-1-AP) Proteintech; https://www.ptgcn.com/products/pictures/pdf/26411-1-AP.pdf; Figure S8m, Figure7i;
Cytokeratin 18 Mouse mAb (sc-6259) Santa Cruz Biotechnology; https://datasheets.scbt.com/sc-6259.pdf; Figure S8m
Vimentin Rabbit mAb (ab92547), Abcam; https://www.abcam.cn/products/primary-antibodies/vimentin-antibody-epr3776-cytoskeleton-marker-ab92547. Figure7i.
Ezh2 (D2C9) XP® Rabbit mAb #5246 l; Cell Signaling Technology; https://www.cellsignal.cn/products/primary-antibodies/ezh2-d2c9-xp-rabbit-mab/5246; Figure 2l, Figure S7e,
Flag Rabbit mAb (AE063) Abclonal; https://abclonalbio.com/Datasheet/Antibodies/AE063.pdf; Figure 3b, 3g, 3h, 3j, 5b, 5f; Figure S4a, S4e, S4h, S4i; S5b, S5c,
anti HA-Tag Mouse mAb (AE008) Abclonal; https://abclonal.com.cn/catalog/AE008； Figure 5b
GFP Rabbit mAb (AE078) Abclonal; https://abclonal.com.cn/catalog/AE078；Figure 3j; Figure S4a
β-Actin Mouse mAb (66009-1-Ig) Proteintech; https://www.ptgcn.com/products/pictures/pdf/66009-1-PBS.pdf; Figure 3b, 3c, 3e, 3f, 5a, 5b, 5f, 5h, Figure S4f, S4h, S4i,
GAPDH Recombinant mAb (81640-5-RR) Proteintech; https://www.ptgcn.com/products/GAPDH-Antibody-81640-5-RR.htm；Figure 2l, 4a, 4b; Figure S3c; Figure S4a, S4h, S5a, S5b, S5c, S7d, S7i, S8a, S8g, S8i,
Lamin B1 Rabbit mAb (#13435) Cell Signaling Technology; https://www.cellsignal.com/products/primary-antibodies/lamin-b1-d9v6h-rabbit-mab/13435； Figure 3k, Figure S4f
HRP-conjugated Mouse Anti-Rabbit IgG Light Chain (AS061) ABclonal; https://abclonal.com.cn/catalog/AS061；Figure 2f, 2g
Protein G PLUS-Agarose: https://www.scbt.com/zh/p/protein-g-plus-agarose
Protein A-Agarose: https://www.scbt.com/zh/p/protein-a-agarose
Anti-DYKDDDDK (Flag) Affinity Gel: https://www.yeasen.com/products/detail/4872
Rabbit Control IgG: https://abclonal.com.cn/catalog/AC005
Mouse Control IgG: https://abclonal.com.cn/catalog/AC011

Eukaryotic cell lines

Policy information about cell lines and Sex and Gender in Research

Cell line source(s)	The human endometrial cancer (EC) cell lines Ishikawa (European Collection of Authenticated Cell Cultures, ECACC; Cat. No. 99040201), AN3CA (American Type Culture Collection, ATCC; Cat. No. HTB-111™) and HEC-50B (Japanese Collection of Research Bioresources, JCRB; Cat. No. JCRB1145), and the human embryonic kidney cell line 293T (ATCC; Cat. No. CRL-3216™) were purchased from ECACC, ATCC and JCRB, respectively. All these cell lines were resuscitated, cultured, and preserved in the Laboratory of Obstetrics and Gynecology, Peking University People's Hospital, and the Laboratory of Science and Technology Building, Peking University Health Science Center. Among them, Ishikawa is a well-differentiated endometrial cancer cell line, HEC-50B is a poorly differentiated endometrial cancer cell line, and AN3CA is an endometrial cancer cell line derived from lymph node metastasis. All endometrial cancer cell lines are derived from female human patients.
Authentication	All cell lines used in this study were authenticated by short tandem repeat (STR) profiling prior to use.
Mycoplasma contamination	Negative
Commonly misidentified lines (See ICLAC register)	NA

Animals and other research organisms

Policy information about studies involving animals; ARRIVE guidelines recommended for reporting animal research, and Sex and Gender in Research

Laboratory animals	Species: Mus musculus (Mouse)<br>Strain : BALB/c Nude (Strain Code: 401; Official Name: CAnN.Cg-Foxn1^nu/Crl<br>Substrain: None (standard Balb/c nude strain)<br>Genetic background: Inbred strain with Foxn1^nu (nude) mutation on the Balb/c genetic background.<br>Sex: Female<br>Age : 4 weeks old<br>Source : Purchased from Beijing Vital River Laboratory Animal Technology Co., Ltd. (Animal Production License No.: SCXK (Beijing) 2019-0010) and the Medical Science and Technology Building of Laboratory Animal Center, Peking University Health Science Center (Animal Production License No.: SCXK (Beijing) 2022-0009); maintained under specific pathogen-free (SPF) conditions.
Wild animals	NA
Reporting on sex	Given postmenopausal EC predominates in females, only female BALB/c nude mice were used, with sex integrated into the study

design.

Field-collected samples	NA
Ethics oversight	The experiment was approved by the Animal Ethics Committee of Peking University People's Hospital (Ethics approval number: 2020PHE094) and IACUC of the Animal Experimental Center of Peking University Health Science Center (Ethics approval number: BCJF0170, LA2019157).

Note that full information on the approval of the study protocol must also be provided in the manuscript.

Plants

Seed stocks	NA
Novel plant genotypes	NA
Authentication	NA
